# The Transformation Experiment of Frederick Griffith I: Its Narrowing and Potential for the Creation of Novel Microorganisms

**DOI:** 10.3390/bioengineering12030324

**Published:** 2025-03-20

**Authors:** Günter A. Müller

**Affiliations:** 1 Biology and Technology Studies Institute Munich (BITSIM), 80939 Munich, Germany; guenter.al.mueller@t-online.de; Tel.: +49-151-25216987; 2 Institute of Media Sociology, Department of Cultural Sciences, University of Paderborn, 33104 Paderborn, Germany

**Keywords:** cellular heredity, horizontal gene transfer, science and technology studies, scientific reductionism, synthetic biology, transforming principle

## Abstract

The construction of artificial microorganisms often relies on the transfer of genomes from donor to acceptor cells. This synthetic biology approach has been considerably fostered by the J. Craig Venter Institute but apparently depends on the use of microorganisms, which are very closely related. One reason for this limitation of the “creative potential” of “classical” transformation is the requirement for adequate “fitting” of newly synthesized polypeptide components, directed by the donor genome, to interacting counterparts encoded by the pre-existing acceptor genome. Transformation was introduced in 1928 by Frederick Griffith in the course of the demonstration of the instability of pneumococci and their conversion from rough, non-pathogenic into smooth, virulent variants. Subsequently, this method turned out to be critical for the identification of DNA as the sole matter of inheritance. Importantly, the initial experimental design (1.0) also considered the inheritance of both structural (e.g., plasma membranes) and cybernetic information (e.g., metabolite fluxes), which, in cooperation, determine topological and cellular heredity, as well as fusion and blending of bacterial cells. In contrast, subsequent experimental designs (1.X) were focused on the use of whole-cell homogenates and, thereafter, of soluble and water-clear fractions deprived of all information and macromolecules other than those directing protein synthesis, including outer-membrane vesicles, bacterial prions, lipopolysaccharides, lipoproteins, cytoskeletal elements, and complexes thereof. Identification of the reasons for this narrowing may be helpful in understanding the potential of transformation for the creation of novel microorganisms.

## 1. Introduction: Artificial Creation of Novel Microorganisms by the Exchange of Genomes

In the course of efforts to create artificial organisms based on transformation, J. Craig Venter and coworkers published a series of important papers in which they reported the following: (i) *Mycoplasma capricolosum*, as intact acceptor bacteria, can be transformed (with the aid of polyethylene glycol) by the complete genome (in the naked and almost protein-free state) of *Mycoplasma mycoides* as donor bacteria [1]. After selection for tetracycline resistance, encoded by the *M. mycoides* chromosome, the transformed *M. capricolosum* harboured the complete donor but lacked any acceptor genomic sequences. Importantly, it did not exhibit any phenotypic differences compared to authentic *M. mycoides*, as revealed by analysis of several independent criteria. (ii) A novel method for the construction of large DNA molecules (e.g., *Mycoplasma genitalium*) from chemically synthesized pieces and from combinations of natural and synthetic DNA fragments by sequential cloning as artificial bacterial chromosomes in *E. coli* and subsequent transformation-associated recombination cloning in the *Saccharomyces cerevisiae* yeast was established [2]. (iii) *M. capricolum* was transformed with intact DNA from *M. mycoides* (with the aid of polyethylene glycol) and resulted in the production of a new viable strain of *M. mycoides*. The latter genome was cloned as a yeast centromeric plasmid, and alterations were introduced into this genome by using yeast genetic systems [3]. (iv) The design, synthesis and assembly of the *M. mycoides* genome, starting from digitized genome sequence information and its transformation into *M. capricolosum* acceptor cells, was reported. It resulted in the creation of new *M. mycoides* cells, solely under the control of the synthetic chromosome (with the markers, deletions and polymorphisms introduced during the construction process), with accompanying expression of the expected phenotypic characteristics and the ability of continuous self-replication [4]. (v) Transposon mutagenesis for the identification of quasi-essential genes required for robust growth of *M. mycoides* was used to produce a synthetic genome (531 kb pairs, 473 genes) under retention of those quasi-essential genes. It was smaller than that of any autonomously replicating cell found in nature and retained almost all genes involved in the synthesis and processing of macromolecules, in addition to 149 genes of unknown function [5]. (vi) Cloning of the entire genome of *M. mycoides* in yeast, the CRISPR/Cas9 system and the yeast recombination machinery were used for the introduction of engineered 16S-rRNA genes [6]. Upon transformation, testing for functionality revealed their resilience to the addition of genetic elements or helix substitutions from phylogenetically distant bacteria. This supported the possibility of construction of simplified artificial *M. mycoides* genomes, thereby answering fundamental questions of (minimal) life (for a review, see Refs. [7,8,9]). (vii) The assembly of a near-complete metabolic network covering 98% of enzymic reactions was achieved by experimental annotation (validation by in vivo transposon mutagenesis), revealing high in vivo essentiality (68%) or quasi-essentiality (92%) compared to in silico essentiality (79%) [10]. This enabled the construction of a model of minimal metabolism in *M. mycoides capricolosum* (JCVI-syn3A) but still left open the function of many genes (of either generic or completely unknown nature). Together, the findings supported specific hypotheses on gene functions and metabolic capabilities and suggested further gene removals aimed at the design of an ultimate minimal cell [10]. (viii) On the basis of those efforts in the development of a minimal bacterial genome capable of achieving metabolic homeostasis, the reproduction of and evolvement to a novel bacterium in an ideally controlled environment have been suggested as the next goals to be tackled. Such a bacterium will rely on a simplified metabolic chart, enabling the production of sufficient energy, as well as of the key components involved, in sufficient amounts [11]. This minimal metabolic network was then coupled to the KEGG database and analysed for its metabolic building blocks. The resulting reaction graph encompassed 80 compounds, 98 reactions, 36 metabolic building blocks and 12 essential reactions necessary for the connectivity of this network. Comparison with the analogous reconstruction of the metabolic network reaction graph of JCVI-syn3.0 revealed a consistent core metabolism despite slightly differing lifestyles. This suggested that the reconstruction of minimal metabolic networks may be the initial step for the creation and manipulation of minimal cells [11]. (ix) The assembly of a 190 kb designer neochromosome housing all 275 relocated nuclear tRNA genes and functioning as a de novo counterpart, in addition to the native complement of *Saccharomyces cerevisiae*, was accomplished by stabilization through the incorporation of orthogonal genetic elements from non-*S. cerevisiae* yeast species [12]. This supported the operation of a potent selective force, as manifested in a spontaneous increase in cellular ploidy. (x) Consequently, the International Synthetic Yeast Project (Sc2.0) was founded to create the first synthetic designer eukaryotic genome [13]. This succeeded in the construction of all 16 nuclear chromosomes of *S. cerevisiae*, as well as a 17th de novo neochromosome encompassing all nuclear tRNA genes. The considerable impact of those findings for the future development of methods of synthetic genomics was claimed [13].

**Figure 1:**
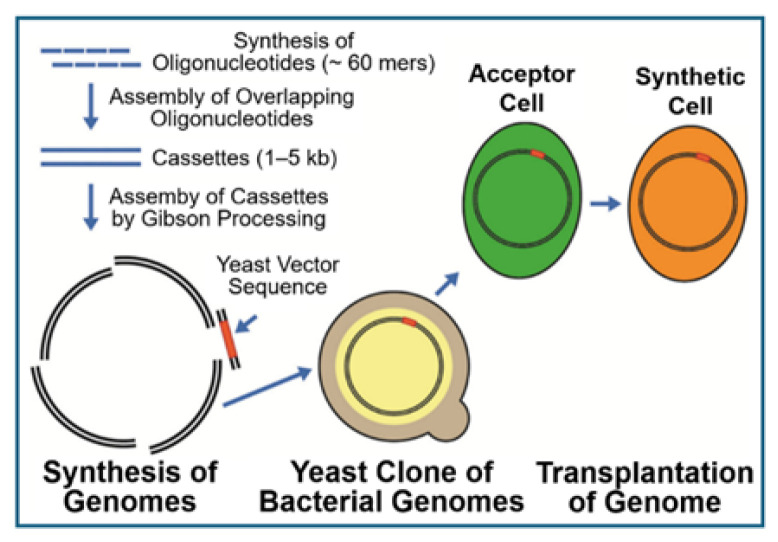
Artificial construction of bacterial cells as a strategy of synthetic biology for the generation of novel organisms as proposed by J. Craig Venter and coworkers. A number of key technologies for the synthesis of genomes have been introduced by the J Craig Venter Institute (JCVI) during the past two decades, which have considerably facilitated the development of bacteria harbouring chemically synthesized genomes. First, the DNA molecules generated with the aid of an inefficient and slow process were limited to max. 32 kb. Subsequently invented methods significantly accelerated the synthesis of larger genomes with greatly improved efficacy and culminated in the first complete chemical synthesis of a bacterial genome (*M. genitalium*) in 2008 [2,15]. This success was based on the assembly of cassettes that had been built up from overlapping synthetic oligonucleotides. Cloning of the bacterial genomic DNA, which encodes a tetracycline resistance marker (for survival in the presence of this antibiotic in the culture broth during transformation of yeast), together with 3–5 kb vector sequences (red), which operate as centromeric plasmids in yeast, was performed to yield the total assembly of those overlapping bacterial sub-genomic DNA sequences. The production process of the high number of bacterial synthetic genomic DNA sequences required for genome transplantation was accomplished by culturing of the yeast cells with the parked synthetic bacterial genome. Following preparation of the synthetic bacterial DNA molecules from yeast at large scale, genome transplantation leads to their introduction into an appropriate bacterial acceptor cell. As a result, the transplanted synthetic genome now manages to direct the genotype and phenotype of the newly emerging cell. For genome transplantation, yeast cells harbouring the completely synthesized bacterial donor genome are encapsulated by small blocks of low-melting-point agarose with zymolyase and proteinase K immersed. These two enzymes provoke the conversion of the yeast cells into spheroplasts, with the bacterial donor genome left in the interior of the agarose blocks. This procedure protects the bacterial genome from shearing forces during the purification process due to its gentle release from the melting agarose. Upon mixing of the released bacterial donor DNA and the bacterial cells (*M. capricolosum*) in the presence of polyethylene glycol (to increase membrane fluidity), as well as CaCl_2_ (to neutralize the charge of the donor DNA), the donor genome is prepared for uptake by the acceptor bacterial cells (albeit at a rather low rate). After growth and division of those acceptor cells (which are of diploid nature for a short period of time only), the addition of tetracycline to the culture results in selection for only bacterial cells that express the tetracycline resistance marker encoded by the synthetic donor genome exclusively [15].

All the above experimental approaches were developed to create the smallest bacterial genome compatible with life based on a top-to-bottom design. By nature, this critically depends on the (minimal) definition of (minimal) life, i.e., replication, metabolism, and heredity [14]. Apparently, the newly constructed partially or completely synthetic bacterial genomes and rearranged yeast chromosomes must “fit” to the corresponding acceptor cells. This adaptation apparently leads to considerable limitations regarding the composition and/or structure of novel proteinaceous components or protein complexes, which originate from—i.e., are encoded by—the donor cell and are introduced by transformation, and the protein counterparts, which are pre-existing in—i.e., already synthesized by—the acceptor cell. This “fitting” could be interpreted in analogy to the key-lock mechanism between substrates and enzymes and may be feasible—presumably only—for closely related bacterial species such as *M. mycoides* and *M. capricolosum*, as well as between orthogonal genetic elements of *S*. *cerevisiae* and other yeast species (Figure 1) (for a review, see Ref. [15]).

Certainly, this limitation is a consequence of the exclusive focus of the presented studies on the modification of genomes that rely on the testing of appropriate predecessor cells for vitality, (minor changes in) phenotype, replication and inheritance. Is there a principal difference between novel, artificial or synthetic bacteria on the one side and typical genetically modified bacteria on the other, which cannot be reduced to the mere extent of the genomic alteration? Importantly, the conception of transformation, which formed the basis of the experimental efforts reviewed above, emerged about a century ago over a period of several decades (for greater details, see Section 2).

As the initial step towards the creation of novel microorganisms, the theoretical considerations and practical reasonings for the assumption that this task can be fulfilled preferentially or even solely with the aid of transformation and the accompanying emphasis on DNA as the sole matter and/or information of inheritance to be transferred between donor and acceptor cells are presented (this manuscript). For this, the scientific beginnings, relevant traditions and experimental systems of the various designs of the original Griffith transformation experiment (1.0–1.1) and the subsequent designs (1.2–l.9) introduced by the scientific successors of Frederick Griffith are discussed with consideration of the underlying historical, economic, political and socio-cultural human and non-human (f)actors. In a next step, a possible strategy for modification of the Griffith transformation experiment and its extensions (2.0–2.X) towards the creation of novel microorganisms will be presented [16].

## 2. The Griffith Transformation Experiment (1.0) and Its Succeeding Designs

### 2.1. The Griffith Experiment in Mice

**Figure 2:**
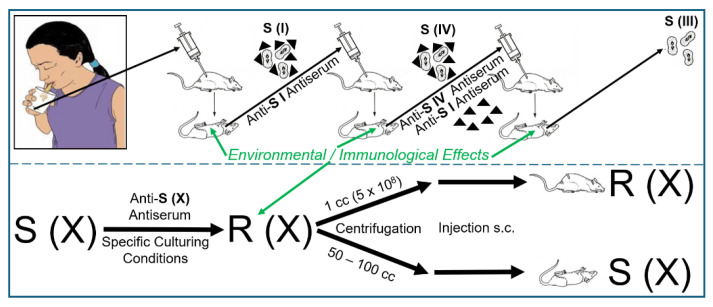
Stability and reversion of pneumococcal serotypes using the Griffith transformation experimental design 1.0 [21]. Upper section: Serotype switching of smooth pneumococci. A sample of the sputum of a patient suffering from lobar pneumonia was injected into a mouse. The propagated bacteria turned out to belong to one of the common serotypes of smooth pneumococci, such as S (I). Another sample of this sputum was mixed with anti-S (I) antiserum to prevent infection of a second mouse by S (I) upon injection of the mixture. This mouse was killed in the course of an infection by pneumococci of a different serotype (S (IV)). This experimental design was repeated with another sample of sputum, which was mixed with both anti-S (I) and anti-S (IV) antisera, and, again, caused the death of the injected mouse due to the emergence of pneumococci of serotype S (III). This experimental setting was used by Griffith to demonstrate that many pneumonia patients exhibit pneumococci of two or more different serotypes. Lower section: The loss or regaining of virulence by pneumococci. S (X) of a certain serotype were grown in the presence of the homologous anti-S (X) antiserum raised against the capsular lipopolysaccharide (LPS) or special agar-containing broth. This led to the loss of the capsule from the former virulent S pneumococci and, concomitantly, the generation of non-virulent encapsulated R (X) pneumococci. They differed considerably in their ability to resynthesize capsular material. Some of them spontaneously regained their virulent character for the mice, whereas others remained more stably in the non-virulent state. The latter, more stable R (X) were tested for serotype switching, which relied on the injection (s.c.) of either 1 cc or 50–100 cc of a fully grown pneumococcal culture into a mouse. Injection of the low dose apparently did not lead to disease, whereas the high dose frequently caused fatal infections by pneumococci, which displayed the same serotype as the original R strain. Apparently, even the stable R (X) pneumococci managed to revert to their S (X) counterparts if injected at high concentrations under appropriate conditions.

One observation that intrigued Frederick Griffith from the beginning of his career as medical microbiologist and clinical immunologist in the early 1920s was that a pneumonia patient could display as many as four to five different serological types of pneumococci [17,18,19] (for a review, see Ref. [20]). He did not believe that the patients had acquired each of these distinct serotypes of pneumococci as a result of separate infections. Nevertheless, it was well known at those times that the dissemination of several distinct serotypes throughout the population occurred during periods of high incidence of pneumonia, increasing the chance and risk of acquiring two or more of them. As an alternative explanation for the simultaneous presence of different serotypes in a single individuum, Griffith proposed the conversion of a given serotype to another one as a consequence of environmental effects or immunological reactions that may occur in the tissues of the infected host or, in his own words [21], “*On a balance of probabilities interchangeability of type seems a no more unlikely hypothesis than multiple infection with four or five different and unalterable serological varieties of pneumococci*”. It was acknowledged then that the occurrence of non-virulent rough (R) forms and virulent smooth (S) forms of a certain type is a spontaneous event and that the same holds true for their reversion to S forms of the same type. Ironically, the type-specific alteration of S to R forms (in the presence of antiserum) and the (type-specific) reversion to S forms has been recognized to, presumably, rely on point mutations of DNA rather than on the recombination of allelic homologues of (long stretches of) DNA insofar as switching between the same serotypes is concerned (e.g., S (IV) to R (IV) to S (IV)). Most importantly, Griffith observed that if alteration of a certain type had occurred, it was solely upon incubation with attenuated (by antiserum or specific culturing) donor pneumococci of the other type to that type, i.e., identical to that of the attenuated donor pneumococci (Figure 2).

The findings of the experiments dealing with the stability and reversion of pneumococci (Figure 2) led Griffith to argue that in the stable R strains, a remnant structure of the original S antigen could be left behind as residual degenerated capsule materials, the amount of which was apparently under the level of detection by serological methods. His fascinating interpretation of the first successful demonstration of the transforming principle relied on an experimental protocol aimed at serotype switching and was based on the assumption of “cycles of states” of the pneumococci [21]: “*It would appear that the type I antigen no longer serves its purpose in the presence of the immune substance formed during convalescence, and the pneumococcus consequently develops its type II side*”. Griffith continued his argumentation that cellular materials, as direct precursors of the S antigen or stimulators of its synthesis, become released in the course of transformation [21]: “*When a strain of this character is inoculated in a considerable mass under the skin, the majority of the cocci break up and the liberated S antigen may furnish a pabulum which the viable R pneumococci can utilize to build up their rudimentary S structure. The amount of S antigen in an R strain, even one only partially attenuated, might not be very large, and it might happen that such an R strain did not liberate in sufficient concentration the stimulating or nutrient substances necessary to produce reversion. It appeared possible that suitable conditions could be arranged if the mass of the culture was derived from killed virulent pneumococci, while the living culture was reduced to an amount which, unaided, was invariably ineffective. There would thus be provided a nidus and a high concentration of S antigen to serve as a stimulus or a food, as the case may be*”.

**Figure 3:**
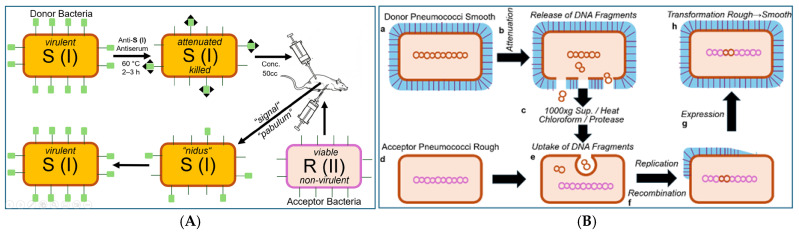
Schematic representation of the original Griffith transformation experiment (design 1.1) in 1928 (**A**; see Ref. [21]) and its canonical interpretation (**B**). (**A**) Donor pneumococci of serotype S (I), corresponding to 50 cc of broth culture, which had been attenuated by incubation with anti-S antiserum (in the presence of chocolate broth) or killed by heat (60 °C, 2–3 h), were injected in parallel with a limited amount of viable R (II) acceptor pneumococci. From the few mice that had been killed after the injection, S (I) pneumococci were isolated and grown in culture to fully virulent S (I). (**B**) Donor pneumococci of the (S) serotype (**a**) release fragments of their genome (brown circles) into the incubation/culture medium due to (partial) lysis and loss of cellular integrity in the course of their attenuation by heat (**b**) and subsequent exposure to the acceptor pneumococci. (**c**) Subsequent researchers [22] subjected the partially lysed donor pneumococci to centrifugation, heat exposure, chloroform extraction and/or protease treatment, etc. (design 1.2–1.X). The released and (eventually partially) purified DNA fragments or plasmids/episomes, possibly intrinsically contained in the donor pneumococci, were then taken up by acceptor pneumococci of the rough serotype (pink DNA circles) (**d**) by molecular mechanisms (**e**), which were characterized in detail. Following recombination of relevant DNA fragments into the genome of the acceptor pneumococci (brown and pink DNA circles) and their replication (**f**), the genes putatively encoded by the DNA fragments become expressed (**g**), including those engaged in the synthesis pathway of the specific LPS of the smooth serotype (pink circles). Consequently, capsule materials of the smooth serotype are formed ((**h**); for a review, see Refs. [23,24,25]). This sequence of events ultimately leads to the conversion of rough to smooth pneumococci and explains the inheritance of a “minor” difference, such as non-virulence vs. virulence, in bacteria.

**Figure 4:**
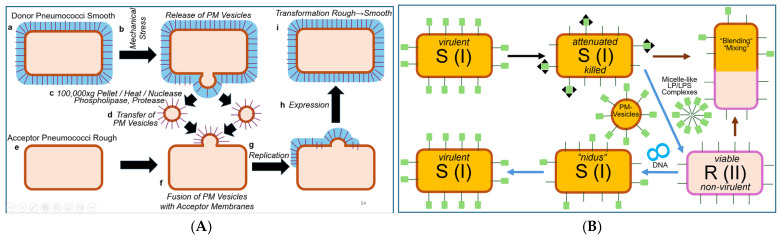
Alternative interpretations of the Griffith transformation experiment (1.1). (**A**) The production of extracellular donor vesicles from the plasma membranes (PM) of donor bacteria (**a**) in the course of specific exocytotic or budding mechanisms have been amply documented (for details, see text below; for a review, see Refs. [26,27,28,29]). PM vesicles that may be released in response to environmental factors, such as mechanical stress (**b**), specifically harbour the LPS determining the smooth phenotype of the donor pneumococci and resist enzymic attacks from both nuclease and protease (**c**). Upon transfer of the PM vesicles from the smooth donor (**d**) to rough acceptor pneumococci (**e**), they fuse with the PM of the latter (**f**). In contrast, the released vesicles are removed by centrifugation (100,000× *g*-pellet); solubilized by extraction with chloroform; degraded by nuclease, phospholipase or protease digestion; or destroyed by heat treatment (80 °C, one hour) (**c**). In the course of fusion of the vesicles donating the smooth LPS (for a review, see Refs. [24,25]) to the PM of rough acceptor pneumococci (**d**) and subsequent replication (**g**) by a molecular mechanism that may resemble the so-called PM memory [16], the acceptor pneumococci gradually switch their phenotype from rough to smooth until completion of transformation (**h**). Complex (e.g., morphological) features of pneumococci other than those mediating the smooth phenotype and not assayed by the transformation experiment (1.0–1.X) may be inherited by PM vesicles (**i**; for details, see below; for a review, see Refs. [30,31,32,33,34,35]). (**B**) Apparently, the R (II) bacteria manage to convert to virulent S (I) and initiate reformation of a capsule, starting from an intrinsic precursor moiety of the acceptor bacteria and completing its final structure in S (I)-typical fashion (blue arrows). Transformation is provoked with the aid of DNA, and/or (putatively) micelle-like lipoprotein/lipopolysaccharide (LP/LPS) complexes and/or PM vesicles. Both the complexes and vesicles could correspond to “signal”, “food” or “pabulum”, as offered by Griffith as an explanation. According to an alternative theoretical option, “blending”, “fusion” or “mixing” of attenuated S (I) and non-virulent R (II) bacteria may result in hybrid pneumococci displaying both (S) and (R) characteristics, with the virulent phenotype dominating (brown arrows).

Consequently, Griffith tried to increase the amount of stimulus, food or pabulum liberated from *S. pneumococci* by the introduction of certain experimental conditions. Inactivation of pneumococci did not create a great problem due to their exquisite sensitivity towards heat, i.e., temperatures above 60 °C for several minutes, as was manifested in the killing of fully grown cultures. However, it was very critical to ensure and to unequivocally demonstrate that each individual pneumococcus of the total population of a given culture was dead. For this, Griffith favoured the procedure of heating the culture at 60 °C for two to three hours, which is considerably longer than the time actually required for pneumococcal inactivation. (Nevertheless, the possibility of the survival of a few bacteria in a given population used for the demonstration of transformation could not be completely excluded. Even 99.99% inactivation would not have been enough to rule out the remote possibility of the continued survival of S instead of the conversion of R to S). Material treated in this way was used successfully for the conversion between specific serotypes of the capsule (Figure 3 and Figure 4).

#### 2.1.1. Bacterial Membrane Vesicles as a Putative Transforming Principle

The liberation of PM and outer membrane (OM) vesicles, constituted by a bilayer membrane of phospholipids and LPS with integrated LP (outer leaflet) and transmembrane proteins (both leaflets) from Gram-positive and Gram-negative bacteria, respectively, with accompanying transfer of a specific subset of the vesicle constituents from the donor to the acceptor cells with functional or (patho)physiological consequences for the latter, in constitutive fashion or in response to certain environmental conditions or signals has been amply documented during the past 15 years [26,27,28,29,30,31,32,33,34,35].

The formation of membrane vesicles was first described by D.G. Bishop and E. Work in the cell-free supernatant of *Escherichia coli* [36]. These are mostly spherical particles with a diameter of 10–500 nm, which are surrounded by a lipidic bilayer membrane. Rarely, they can take on an elongated or elliptical shape. In their interior, i.e., the lumen, they can contain metabolites, soluble proteins, membrane proteins, polysaccharides and nucleic acids, which are collectively referred to as cargo. Sometimes, they also contain misfolded proteins, antibiotics or metal ions, depending on the growth conditions of the microorganisms. Various terms have been created to designate these membrane vesicles according to their origin: the term PM vesicles, by definition, refers to the vesicles released by Gram-positive bacteria and archaea, while the term OM vesicles refers to the vesicles released from the OM of Gram-negative bacteria.

While membrane vesicles were initially considered to be random products of cell lysis, it is now known that they are secretion products of living cells. Since their discovery, they have been intensively studied in bacteria, in particular [37], where they play an important role in horizontal or lateral gene transfer. OM vesicles have a diameter of 20–230 nm and are formed by both pathogenic and non-pathogenic bacteria. Their membranes consist of phospholipids, proteins of the outer and inner membranes, and lipopoly- or lipo-oligosaccharides (LPS). Mass spectrometric studies have identified more than 3500 different proteins in OM vesicles. As cargo, they can contain nucleic acids, ions, metabolites and signalling molecules. DNA molecules can be located in the lumen or bound to the surface. RNA molecules have also been detected in the lumen. How the nucleic acids get into the lumen is still unknown.

Three models are discussed for the mechanism of formation of OM vesicles, the first of which is (i) Loss or rearrangement of covalent bonds between the OM and the underlying peptidoglycan layer. Due to this process, the OM grows faster than the cell wall, which leads to its bulging and, thus, to the initiation of vesicle formation. (ii) The accumulation of peptidoglycan fragments or misfolded proteins in periplasmic space produces turgor pressure on the OM, followed by its bulging and subsequent constriction. The final possible mechanism is (iii) the enrichment of molecules that cause bulging or blebbing of the OM, such as quinolone PQS or certain LPS of the OM.

The formation of OM vesicles has been demonstrated under different growth conditions and in different natural biotopes in all pathogenic and non-pathogenic bacterial species studied so far. The following functions have been discussed: horizontal gene transfer, formation of biofilms (mucus layer formed by bacteria on surfaces with embedded microorganisms), stress response, transfer of toxins and other biomolecules, killing of competing microorganisms, resistance to antibiotics, attachment to host cells and modulation of the immune response. Interestingly, OM vesicles can also increase the resistance of bacteria to phages, provided that they have receptors for phages on their surface. The phages then bind to these receptors and inject their genetic material into the vesicles. Since it cannot multiply there, the infection cycle is interrupted, and the bacteria are spared. In addition, publications in recent years have shown that OM vesicles can act excellently as vaccines.

The cell envelope of Gram-positive bacteria consists of a PM and a multilayer peptidoglycan layer, i.e., the cell wall. The PM is an asymmetrical phospholipid bilayer. The outer layer contains teichonic acid, lipoteichonic acid and phospholipids, and the inner layer contains phospholipids and cardiolipin. The production of PM vesicles is not common in Gram-positive bacteria. So far, they have been described in *Staphylococcus aureus*, various *Bacillus* species, *Streptomyces coelicolor* and some other species [38]. These vesicles have a diameter of 20–250 nm and contain cytoplasmic PM and extracellular proteins. Proteins include enzymes that play a role in the degradation of peptidoglycan and antibiotics, as well as virulence factors (anthrolysin, anthrax toxin, haemolysine and lipases). In *Staphylococcus aureus*, 90 different proteins have been described in PM vesicles.

How the PM vesicles overcome the thick peptidoglycan layer after their formation and whether cell wall-modifying enzymes play a role in this are still being discussed. There are three non-mutually exclusive theories as to how PM vesicles of Gram-positive bacteria can pass through the thick cell wall superimposed on PM. (i) The PM vesicles could be forced through the cell wall by the turgor pressure. The pore size and thickness of the cell wall would regulate this process. (ii) Cell wall-modifying enzymes were found together with the PM vesicles and could change the structure of the cell wall in such a way that the PM vesicles could pass through more easily. (iii) Protein channels could facilitate the passage of PM vesicles across the cell wall.

In 1989, it was published for the first time that linear and circular DNA molecules in OM vesicles are excreted by donor *Neisseria gonorrhoeae*. These vesicles fuse with cells of the same species, and the DNA thereby enters the cytoplasm of these acceptor cells. In 2000, it was reported that DNA from an *E. coli* strain can be transferred to a *Salmonella enterica* strain via OM vesicles. This was confirmed by an antibiotic resistance marker that was transferred from the donor to the acceptor cell, where it was expressed (for a review, see Refs. [39,40]).

How can DNA get into PM vesicles? Five possibilities have been described so far. The first is (i) the cytoplasmic pathway, where components of the cytoplasm, including DNA, enter the PM vesicles during their formation. The second possibility is (ii) the periplasmic pathway, where the DNA first passes from the cytoplasm into the periplasmic space, then into the PM vesicles. Under (iii) the extracellular pathway, parts of PM vesicles combine after being released from bacteria and can take up DNA during assembly. Another option is (iv) the uptake of DNA after cell death, whereby free DNA molecules can bind to the outside of PM vesicles after the lysis of bacterial cells. Finally (v) phages could inject their DNA directly into PM vesicles if they express the corresponding phage receptor on their surface. DNA molecules up to 370 kb in size have been detected in PM vesicles so far, but overall, smaller fragments are more common. The DNA fragments are chromosomal fragments, as well as plasmid and phage DNA.

PM vesicles allow for the transfer of DNA, even in bacterial species that do not contain competence genes, such as *E. coli*. In contrast, gene transfer in bacterial species with competence genes, such as in *Acinetobacter baylyi* and *Thermus thermophilus*, only occurs if they are also expressed and, therefore, the competence machinery is present in the cell. The mechanisms of DNA transmission via PM vesicles are largely unclear. The same applies to RNA molecules (mRNA, rRNA, tRNA and sRNA, where sRNA stands for “small RNA”, i.e., small RNA molecules with a mostly regulatory function). Various possibilities have been discussed with respect to how PM vesicles can bind to the cell surface, followed by their lysis or internalization. In the next step, the DNA molecules must recombine with the chromosome so that the genes can be passed on stably. This has been demonstrated for antibiotic resistance genes, metabolic properties and virulence genes. In the last step, the DNA must be absorbed stably. In the case of chromosomal fragments, they must recombine with the chromosomal DNA. This requires identical or almost identical DNA sequences in both molecules and, moreover, is responsible for the necessity of “fitting” between the nucleic acid components transferred from donor to acceptor bacteria, in addition to the proteinaceous components (as already mentioned above). When transferred, complete plasmids can replicate directly if their replication genes are successfully expressed.

Our own genome contains about 300 genes of bacterial origin. What mechanism was used to transfer these from bacteria to human cells? For them to be passed on from generation to generation, they must have entered the germline. Theoretically, this could have been done via one of the three classical mechanisms for DNA transfer, i.e., transformation, chromosome transfer (conjugation) or transduction by phages or episomes (for further discussion, see below). Another possibility is the fusion of germline cells with PM vesicles.

During transduction, phages transfer bacterial genes from one bacterial cell to another of the same species. Apparently, the phages recognize a species-specific receptor on the surface of the bacteria, bind to it and inject their DNA into their cytoplasm. There, the phage DNA is replicated and packaged into newly synthesized phage particles. The infection process often leads to fragmentation of the bacterial DNA, and these fragments can be individually packed into the phage particles instead of the phage DNA. In this way, parts of the bacterial DNA can be transferred to other bacterial cells and recombine with the existing chromosomal DNA.

A few years ago, scientists at the Hebrew University in Jerusalem were able to show how transduction can also work in cells with a defective phage receptor. They incubated *Bacillus subtilis* cells displaying the YueB receptor, which is recognized by the SPP1 phage, on their surfaces, together with bacteria that lacked this receptor and were, therefore, resistant to this phage. Surprisingly, genes were nevertheless transferred from the sensitive to the resistant cells [41]. As an underlying mechanism, the researchers discovered that PM vesicles from sensitive bacteria fused with those of resistant bacteria and, thus, transferred the YueB receptor to them. Apparently, phages can recognize bacterial cells with matching surface receptors and bind to them. They then inject their DNA into the bacterial cell and let it produce new phage particles. During the production of PM vesicles by the phage-sensitive bacterium, phage receptors can be incorporated into the vesicle membrane. If these PM vesicles fuse with a phage-resistant bacterium, the receptors are transmitted transiently. The formerly resistant bacterium can then also be infected by SSP1 phages in order to obtain chromosomal DNA from sensitive bacteria.

In further experiments, these scientists were able to prove that cells from two other *Bacillus* species, i.e., *B. megaterium*, and *B. cereus*, can be transduced via this mechanism, as long as they contain the phage receptor on their PM [41]. In this context, the question arises as to whether bacterial genes can also be spread across species in natural biotopes by this mechanism.

#### 2.1.2. Extracellular LP/LPS Complexes as a Putative Transforming Principle

In contrast to the production of PM and OM vesicles by Gram-positive and Gram-negative bacteria, respectively, the liberation of LP/LPS complexes, constituted by mixed micelles of (lyso)phospholipids and LPSs with intercalated LPs, from donor bacteria and subsequent transfer of LPs and/or LPSs to acceptor bacteria with functional consequences is hypothetical (for further discussion, see Ref. [16]). Interestingly, this process may correspond to the release from and the intercellular transfer between mammalian donor and acceptor cells of micelle-like complexes consisting of (lyso)phospholipids, cholesterol and glycosylphosphatidylinositol-anchored proteins [42,43] (for a review, see Refs. [44,45,46,47]) or lipid-modified proteins [48,49] (for a review, see Ref. [50]), which have been increasingly reported over the past two decades. Importantly, heritable phenotypic switching of the acceptor cells, e.g., stimulation of lipid synthesis in adipocytes and glycogen synthesis in erythroleukemia cells, was demonstrated upon successful transfer of LP/LPS complexes [51,52]. This supports the operation of those complexes as non-DNA matter of inheritance in eukaryotes under specific conditions [53,54]. In addition to OM or PM vesicles and micelle-like LP/LPS complexes, the “blending”/“mixing”/“fusion” of attenuated/killed S (I) and viable non-virulent R (II) represents a theoretical possibility (for further discussion, see Ref. [16].

Early studies [55,56,57] hinted at the polysaccharide nature of the pneumococcal S antigen (here, depicted as green square), which was confirmed later in the course of final elucidation of its detailed structure (for a review, see Refs. [58,59,60,61]).

It is conceivable that both attenuation (by antiserum) and heating (60 °C), i.e., experimental design 1.0, are compatible with the release of PM vesicles or micelle-like LP/LPS complexes from the pneumococci as a consequence of their physical damage or even lysis. Concomitantly, the structural and functional integrity of both the vesicles and the complexes, in general, and of their constituent LPS, LP and proteins, in particular, may be retained. This would justify the necessity for pretreatment of the donor bacteria in the donation process and provide an explanation for the underlying molecular mechanism of acceptance in the course of fusion of the PM vesicles with or integration of the LP/LPS complexes into the PM of the acceptor bacteria. The putative preservation of the integrity and functionality of the PM vesicles or micelle-like LP/LPS complexes regarding their transforming activity on acceptor pneumococci under concomitant maintenance of their generation by and liberation from donor pneumococci in response to attenuation or heating awaits experimental clarification in the future.

As a control to exclude the possibility that neither escape from attenuation, nor heating procedures nor revival of the attenuated or killed S bacteria was a sound explanation, mice were administered a high concentration of inactivated S pneumococci in the absence of added viable R bacteria. In no case were the animals killed by infection or provided living S pneumococci upon culturing. Furthermore, Griffith performed analogous transformation experiments using other pneumococcal strains. Pneumococci of serotype R (I) were transformed into serotype S (II) or (III), and pneumococci of serotype R (II) were transformed into S (I) or (III). Thus, transformation of acceptor bacteria and switching of their serotype were critically dependent on the use of donor bacteria and corresponded to the serotype of the latter.

This epochal outcome of his transformation experiments was commented upon by Griffith as follows [21]: “*When the R form of either type is furnished under suitable conditions with a mass of the S form of the other type, it appears to use that antigen as pabulum from which to build up a similar antigen and thus to develop into an S strain of that type*”. Those materials mentioned by Griffith, i.e., pabulum or signals, could be constituted of micelle-like LP/LPS complexes or PM vesicles (see above), both of which had not been identified or characterized in Griffith’s time, nor had their exchange as a consequence of “blending”, “mixing” or “fusion” of two distinct (but related) populations of bacteria, such as between R and S pneumococci, been considered as a theoretical possibility by Griffith and his successors.

Furthermore, Griffith resisted presenting any explanation for the extremely remarkable finding that the switching in serotype, i.e., transformation, was permanent, i.e., that after the initial building-up of a capsule of a new S serotype by the operation of pneumococci of the R serotype, it continued to operate as such during culture through countless generations. Apparently, something had happened that managed to manifest and perpetuate the alteration in capsule serotype and virulence vs. non-virulence. According to the state of knowledge in 1928, it had to be concluded that each appropriate R strain seemed to be convertible to *S. pneumococci* of any selected serotype if the experimental conditions of the mouse treatment including the pneumococcal infection followed the protocol introduced by Griffith.

In conclusion, the scientific merit of Griffith with regard to introducing experimental designs 1.0 and 1.1 predominantly relied on two facts: The first is (i) the use of appropriate methods to damage, i.e., to attenuate, the S-donor pneumococci in such a way as to achieve balanced strength (by their incubation in the presence of chocolate broth, together with anti-S antiserum or moderate heating). As a result, they lost their ability to replicate and grow (and, therefore, did not interfere with the expansion of the transformed acceptor cells). Concomitantly, they maintained their ability to release the transforming principle into the culture medium in a form compatible with its acceptance by R pneumococci. Secondly, (ii) the selection of a small phenotypic difference (i.e., virulence vs. non-virulence caused by an LPS) rather than a major (e.g., complex morphological or metabolic) feature (see below) was amenable to transfer from donor to acceptor pneumococci according to the transforming principle.

An interesting question for the philosophy of science might be whether the introduction of this experimental system of this design solely relied on (i) serendipity (i.e., virulence of pneumococci as a character due to focus on pneumonia by chance), as has previously concluded by M.R. Pollock [62] (“*Griffith seemed to have had little idea of how this transformation came about, nor even of its great and ultimate significance. His discovery was a classic instance of pure serendipity*”) and R. Olby [63]; (ii) previous observations and hypotheses derived thereof (i.e., use of S antiserum in pneumonia therapy and observation of the recurrence of virulent strains), as has been acknowledged by previous researchers investigating serotype switching [64,65]; (iii) variation and differential reproduction of experimental conditions (i.e., variation of the method of attenuation of donor pneumococci from incubation with anti-S antiserum to moderate heating), as initially been suggested by H.-J. Rheinberger (in a different context) for the interpretation of experimental systems as the discontinuous production of inscriptions and traces [66,67]; or (iv) rational thinking, hypotheses-driven research derived thereof and empirical testing of the hypothesis (i.e., conversion of non-virulent to virulent pneumococci by theoretically thinking about the necessity of only contact or presence of both in the same environment), as has been claimed by representatives of the verification (logical positivism) or falsification (critical rationalism) paradigm of scientific theories [68,69].

### 2.2. The Griffith Experiment in the Test Tube

**Figure 5:**
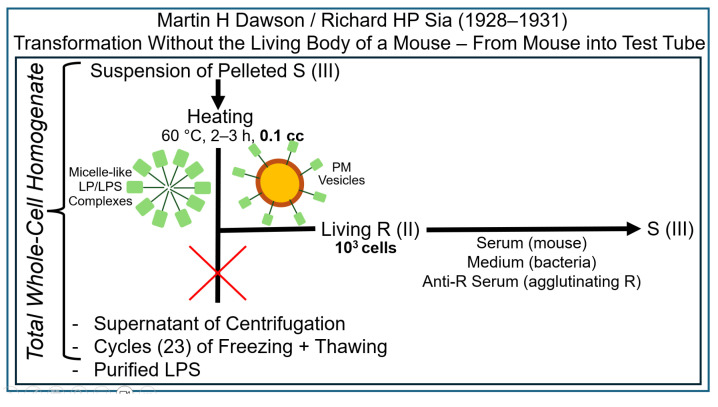
Elimination of the need for passage through the mouse, as well as of the requirement of intact donor bacteria, for the demonstration of transformation (experimental design 1.2; see Refs. [70,71,72,73,74]). A small number of heat-killed pneumococci of serotype S (III), representing a total whole-cell homogenate of the donor bacteria, was incubated (48 h) with a small number of living acceptor pneumococci of serotype R (II) in the presence of mouse serum (to mimic the animal environment), bacteriological culture medium and a minute concentration of anti-R antiserum (to agglutinate the R pneumococci). This resulted in the emergence of pneumococci of serotype S (III) in some experiments, which was demonstrated by subsequent culturing and immunological analysis of the blood of infected mice. In contrast, the supernatant of a high-speed centrifugation of the total homogenate that had been prepared from the heat-killed S (III) pneumococci or the total homogenate generated by up to 23 cycles of freezing and thawing or preparations of the specific LPS purified from the total homogenate did not support the conversion of R (II) into S (III) pneumococci. PM vesicles or micelle-like LP/LPS-complexes are thought to be liberated from the pelleted, then resuspended S (III) donor pneumococci into the total (whole-cell) homogenates and to retain their putative transforming activity in the course of moderate heating (as is certainly the case for DNA and LPS) but may be eliminated from the supernatant of a high-speed centrifugation (PM vesicles), as well as by multiple cycles of freezing and thawing. Moreover, purified LPS turned out to be ineffective in eliciting transformation.

In a remarkable series of papers published from 1928 to 1931 [70,71,72,73,74], Martin Dawson and his coworker, Richard Sia, demonstrated that passage of donor bacteria through a(n) (intact, viable) mouse and the intactness of the organism are not prerequisites for the transformation of acceptor bacteria to S pneumococci, as revealed by both in vitro immunological characterization in a test tube of an LPS of type S and in vivo demonstration of lethality in some mice after infection (Figure 5).

In fact, Dawson and Sia failed in the demonstration of transformation of pneumococci in the absence of total (whole-cell) homogenate prepared from the donor bacteria (initially called a whole-cell vaccine by Dawson). The failure of repeated freezing and thawing (under aerobic conditions, as mentioned in short by Griffith in his original report [21]), as well as of preparation of a homogenate by removal of crude particulate materials in the course of a low speed-centrifugation (i.e., commonly used rather than total whole-cell homogenates), could later easily be explained by the induction of autolytic enzymes. This was presumably a consequence of the disruption of the cellular architecture, causing the complete destruction of the transforming principle. Alloway’s interpretation of the results of the above findings on transformation was expressed as follows [75,76]: “*In considering the nature of the mechanisms by which transformation of type is effected two possibilities present themselves: either a latent attribute of the R cell may be stimulated by its association with the S vaccine, or the organisms may acquire a new property from the vaccine. The former conception involves the assumption that all pneumococci possess the latent capacity of elaborating any one of the known varieties of specific polysaccharide associated with S. The latter hypothesis suggests the possibility that, at times, certain attributes of bacteria may be transferred from organisms of one type to those of another type of the same species.*”

**Figure 6:**
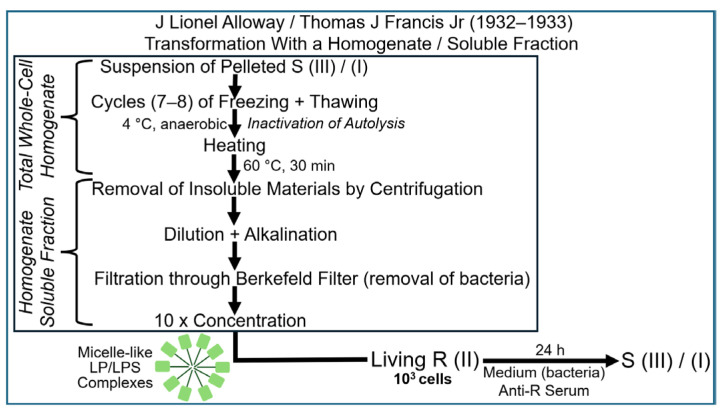
Demonstration of the operation of a typical homogenate/soluble fraction supporting transformation (experimental design 1.3; see Refs. [75,76]). Total (whole-cell) homogenates were prepared from pneumococci of the S (III) or S (I) serotype by using a limited number of cycles of freezing and thawing (rather than heating)—a considerably reduced number of cycles in comparison to that used by Dawson and Sia (see Figure 5). After heating of the total (whole-cell) homogenate and centrifugation for removal of crude particulate materials, the resulting supernatant was diluted and passed through a bacterial filter (made of porcelain and named a Berkefeld filter after its inventor). This procedure caused the removal of any bacteria while enabling the passage of any soluble materials (after adjustment of the pH of the homogenate to an alkaline level, thereby avoiding non-specific adsorption of its components by the porcelain material), thereby excluding the possibility that an occasional viable S pneumococcus in the homogenate could be responsible for the supposed positive transformation event. The filtered homogenate was concentrated about ten-fold and incubated with a small number of viable R (II) pneumococci in the presence of bacterial medium and anti-R antiserum.

On the basis of the information about the experimental set-up that enabled transformation using total (whole-cell) homogenates of the donor pneumococci and that had been published in a preliminary report by Dawson and Sia in 1930, J Lionel Alloway tried to obtain subcellular fractions from similarly prepared total homogenates that did maintain their capability of transformation. The results of his efforts were reported in 1932 and 1933 [75,76], clearly revealing the methodological similarity to the approach initially introduced by Dawson and Sia; however, the author did not mention the numerous wrong assumptions and massive frustrations (Figure 6).

Micelle-like LP/LPS complexes were thought to be liberated from the pelleted, then resuspended S (III) donor pneumococci into the total (whole-cell) homogenates in the course of multiple cycles of freezing and thawing, followed by moderate heating and subsequent conversion of this homogenate to a soluble fraction via a series of steps. This fraction was assumed to retain its putative transforming activity (as was certainly the case for DNA). PM vesicles would be completely eliminated from the soluble fraction of the homogenate during the multi-step process of its preparation.

**Figure 7:**
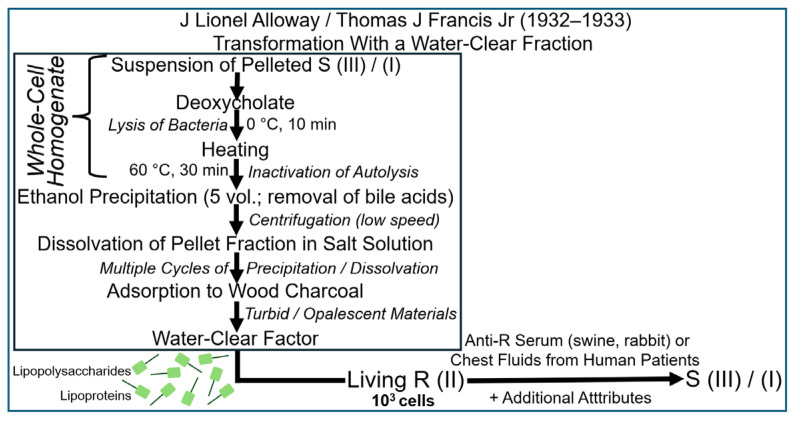
Demonstration of the operation of a water-clear fraction supporting transformation (experimental design 1.4; see Refs. [75,76]). Total (whole-cell) homogenates were prepared from pneumococci of the S (III) or S (I) serotype by lysis with sodium deoxycholate and subsequent heating. After precipitation of most of the material released from the pneumococci with ethanol but leaving behind the bile salt, a pellet of a low-speed centrifugation was dissolved in salt solution. The material contained in this solution was precipitated and redissolved again without significant loss of transforming activity. Following treatment with powdered wood charcoal, the opalescent and weakly turbid solution was converted into a water-clear factor, putatively containing dispersed LP and LPS in detergent solution. Upon incubation with a small number of viable R (II) pneumococci in the presence of anti-R antiserum, this factor was capable of transforming them into S (III) or S (I) pneumococci, respectively. PM vesicles, as well as micelle-like LP/LPS complexes, were certainly eliminated from the water-clear factor in the course of the multi-stage preparation of the whole-cell homogenates, encompassing detergent solubilisation and subsequent purification of the factor. LPS and LP solubilized with deoxycholate may have survived this enrichment procedure (as was apparently true for DNA).

This experimental configuration resulted in the emergence of S (III) or S (I) bacteria at varying efficacies, which could only be explained by transformation of acceptor pneumococci using a soluble fraction derived from the total (whole-donor cell) homogenate, which Alloway had apparently succeeded in introducing first. In fact, Dawson and Sia came to similar conclusions about the necessity of reducing the number of thawing and freezing cycles in order to obtain the transformation principle in the active state in solution. However, Dawson terminated ongoing efforts and intentions to publish in deference to Alloway. Subsequently, in order to improve the admittedly low efficacy, reproducibility and reliability of the transformation activity of the soluble fraction, Alloway introduced several modifications of the experimental protocol aimed at minimizing the loss of transforming activity during disruption of the pneumococci [75,76]. The most critical modification during the lysis process was certainly the use of bile salt sodium deoxycholate, which had commonly been used for the solubilization and classification of pneumococci instead of repeated freezing and thawing. Pneumococci have long been known to become totally destroyed in the course of suspension in bile based on microscopic examination. Alloway took advantage of this efficacy of bile in lysing pneumococci and started the procedure at 0 °C for 10 min, with subsequent warming of the suspension to up to 60 °C to prevent autolysis by intrinsic bacterial enzymes. This protocol took considerably less time than that involving freezing and thawing (Figure 7).

An additional modification was introduced by Alloway concerning the assay system for the transforming principle. This obviously improved the effectiveness and reliability of the soluble water-clear fractions to yield a positive outcome and included the addition of chest fluid or other pathological fluids from human patients. Like anti-R antisera from rabbits or swine, those fluids were also capable of causing agglutination of R pneumococci but seemed to harbour other attributes, which made them superior to the use of animal sera; moreover, they were cheap and available in large quantities.

In conclusion, the experimental procedure and the assay, both of which were completed and published in 1933 [76], unequivocally demonstrated the involvement of a soluble water-clear factor contained in the total homogenate of donor pneumococci in the transformation of acceptor pneumococci to the serotype displayed by the former. However, this factor seemed to operate in an inconsistent and sometimes unpredictable fashion. The problem with reproducibility necessitated the development of a reliable test system for assaying subcellular fractions with high precision and accuracy, a prerequisite for the purification of the transforming principle and the characterization and identification of its nature. In fact, this strategy was subsequently followed predominantly at the Rockefeller Institute in New York (see below).

### 2.3. The Griffith Experiment with Subcellular Fractions

**Figure 8:**
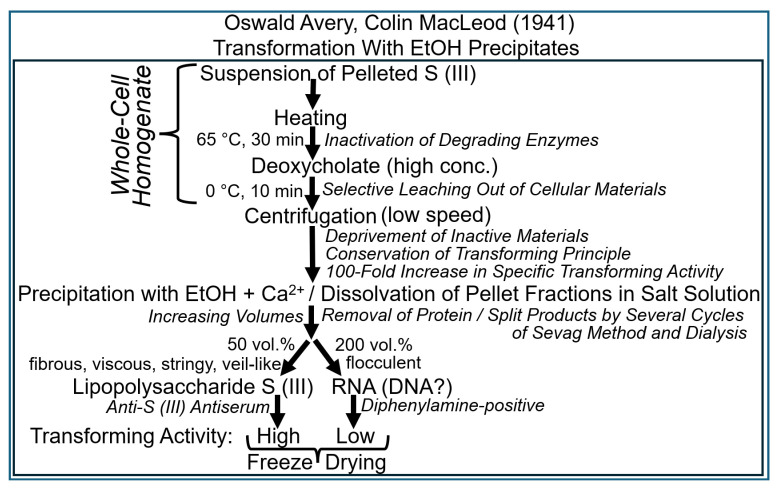
Demonstration of the operation of EtOH precipitates supporting transformation (experimental design 1.5; see Ref. [77]). A suspension of pneumococci that had been collected by centrifugation was heat-killed and extracted in the presence of deoxycholate. After removal of the cells by centrifugation, the extract was precipitated by increasing concentrations of ethanol in combination with Ca^2+^ (Sevag method, see Ref. [78]). The redissolved pellet fractions were dialyzed, then displayed an either fibrous (25% ethanol, final conc.) or flocculent (66% ethanol, final conc.) appearance. The two distinct precipitates were tested for the presence of both LPS of serotype S (III) or nucleic acids and transforming activity before stockpiling by freeze drying. PM vesicles, as well as micelle-like LP/LPS complexes, were certainly eliminated from the water-clear factor in the course of the multi-stage preparation of the whole-cell homogenate, which included detergent solubilisation and subsequent fractionated precipitation with ethanol. Detergent-solubilized LPS and LP may have been eliminated from the transforming principle in the course of fractionated ethanol precipitation (as was apparently not true for DNA).

Colin MacLeod commented on the precipitation procedure he had introduced after having joined the laboratory of Oswald Avery at the Rockefeller Institute as follows [77]: “*It has been noticed previously that when alcohol is added to an extract, the precipitates formed are of 2 kinds–a. coming out at about ½–¾ volumes of alcohol is stringy, veil-like; b. flocculent at higher concentrations of alcohol. Therefore, the attempt is made to obtain these 2 gross fractions separately*” (Figure 8).

In this type of experiment, most of the transforming activity and most of the S (III) LPS was enriched in the former type of fibrous precipitate and—apparently—most of the RNA in the flocculent fraction. Importantly, a test with organic chemical diphenylamine was introduced at this stage of experimentation—a test relying on the presence of deoxyribose in the sample. It was found that both before and after digestion with ribonuclease and subsequent dialysis, the extract yielded a clear-cut positive reaction. This was manifested by the formation of a china-blue colour, which is characteristic of deoxyribose and, as a consequence, also of DNA. MacLeod interpreted this outcome as follows [79]: “*Thus it would appear as though these transforming extracts may contain a little desoxyribonucleic acid in addition to the large amount of ribosenucleic acid present*.”

It had previously been found that the operation of an enzyme in the synthesis of glycogen was critically dependent on the presence of some glycogen molecules that had been prefabricated during the previous reaction cycle. This dependence led to the conclusion that this enzyme needs to be primed by (a part of) the end product that is produced by it in the course of glycogen synthesis. Avery and MacLeod explained the somewhat ambiguous results with retention of transforming activity in the fibrous fraction containing the LPS of the S serotype. In their differential fractionation experiments, they observed the possibility that something to do with the type of a primer, which was precipitated by 25% ethanol, could mediate transformation [80].

**Figure 9:**
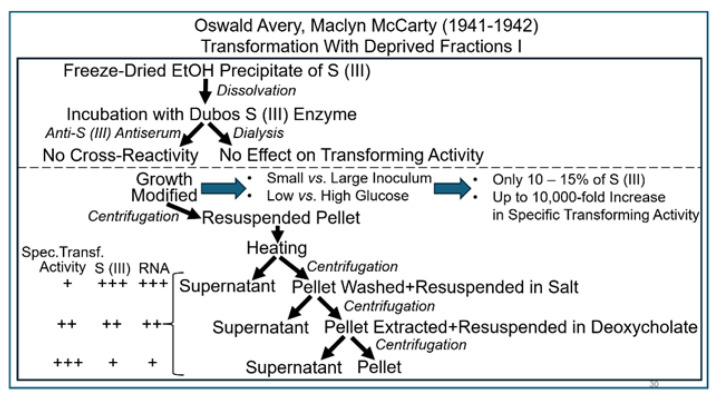
Exclusion of S (III) LPS and RNA as the transforming principle (experimental design 1.6.1; see Ref. [80]). Upper section: An ethanol-precipitated, freeze-dried, redissolved preparation of the S (III) LPS was treated with Dubos SIII enzyme [81]. The digested materials did not display any serologically detectable S (III) antigen and, concomitantly, did not affect the transforming activity of the enriched preparation of S (III) LPS, which, per se, turned out to account only for slightly above background levels. Lower section: Pneumococci grown at low initial inoculum and glucose levels were recovered by centrifugation and, after resuspension, subjected to heat killing. Following centrifugation, the pelleted cells were washed with salt solution and re-centrifuged. This cell pellet was submitted to extraction by deoxycholate and re-centrifuged again.

Although they did not completely abandon their opinion that the transforming principle was not identical to that of the S (III) LPS, Avery and MacLeod did not exclude the possibility that enzymes become activated by the transforming principle. Such enzymes may manage to initiate the production of the S (III) LPS, resulting in the formation of a functional capsule of the S (III) serotype. In analogy with glycogen priming, the initiation of this reaction cascade leading to the complete LPS was assumed to critically depend on priming by some residual S (III) molecules. However, Avery and coworkers were not convinced about previous efforts aimed at the (quantitative) elimination of an eventual function of S (III) LPS as the (constituting part of the) transforming principle. For instance, both Rogers (1933) and MacLeod (1935) tested the impact of the glycogen-degrading so-called Dubos SIII enzyme on the capability of crude extracts to transform pneumococci and did not recognize any difference between before and after the treatment [80]. Consequently, it was the first job of Maclyn McCarty to resolve this issue in September 1941 [80]. Thus, he tried to clarify the question as to whether the availability of a small amount of S (III) LPS in the transforming principle was critical, acting as a primer or template for the transformed pneumococci to initiate the production of new LPS entities as the building blocks of a functional capsule (Figure 9).

A test of the individual fractions demonstrated considerable transforming activity in the heat-killed pneumococci, as well as in the salt wash; however, the transforming activity was significantly higher in the latter compared to the former. However, the highest specific transforming activity was contained in the final deoxycholate extract, where any losses caused by the fractionation procedure were more than compensated for by a marked decline of the amount of S (III) LPS, as well as RNA, compared to that displayed in the supernatant of the heat-killed cells.

**Figure 10:**
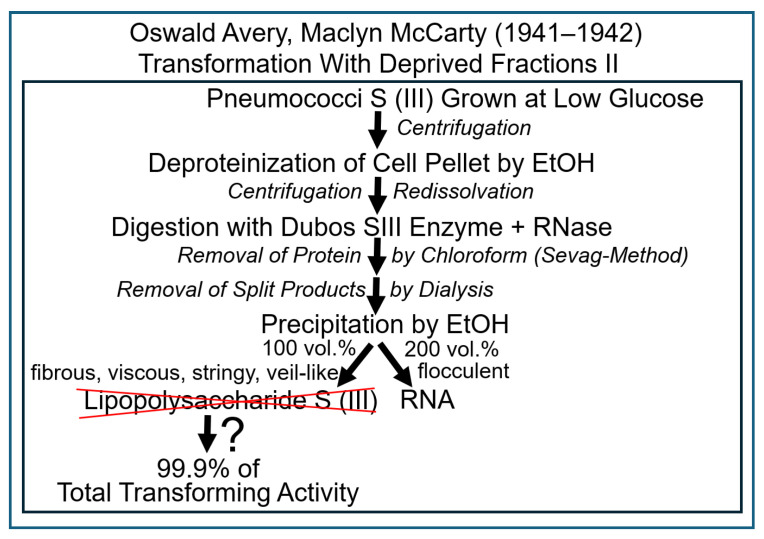
Pneumococci grown at low initial inoculum and glucose levels were recovered by centrifugation and, after resuspension, subjected to deproteinization (experimental design 1.6.2; see Ref. [80]). Following redissolvation, the deproteinized materials were digested by both SIII Dubos enzyme [81] and ribonuclease, followed by removal of the added enzymes by repeating the Sevag method [78] and of the cleavage products of the digestion by dialysis. After the addition of increasing volumes of ethanol, precipitates of different characteristics became visible in sequential fashion from 50% to 66% ethanol (final conc.)—first as fibrous precipitates and later as more flocculent precipitates. Strikingly, the major portion of the total transforming activity was recovered with the fibrous fraction.

Subsequent experimentation using repeated cycles of salt washings of the pneumococci prior to deoxycholate extraction and quantitative titration led to the conclusion that more than 90% of the total transforming activity was recovered with the deoxycholate fraction (Figure 10).

Consequently, it was concluded that a function of S (III), as a sort of primer, does not represent a prerequisite for transformation. Nevertheless, the presence of S (III) LPS in large amounts could have interfered with future efforts to isolate and identify the transforming principle due to its residual activity, which was most likely based on a sort of molecule different from S (III). Therefore, in the next set of experiments, the researchers attempted find a strategy to reduce or even to get rid of S (III), as has previously been demonstrated successfully with protein. Since Dubos SIII enzyme had not been available in the amounts required for treatment of large volumes of pneumococcus broth at the time, the culturing conditions were modified to accelerate growth by using a smaller initial inoculum and at a low glucose concentration, which were known to be associated with considerably delayed and dampened synthesis of cell wall materials such as LPC, respectively. This procedure resulted in a considerable enrichment in the transforming principle, as revealed by the increase in specific activity.

Next it was tested whether washing of the pneumococci before extracting them with deoxycholate would lead to a reduction in the amount of the S (III) LPS associated with their surfaces and possibly even co-fractionated with RNA. At this point, no diphenylamine test for the demonstration of deoxyribose in the fractions was applied, and the presence of DNA was not taken into serious consideration by Avery and McCarty. They exclusively paid attention to methods for the sensitive detection and demonstration of the absence of protein, RNA and S (III) LPS. Nevertheless, this experiment marked the start of a period during which their focus shifted, with increasing sharpness, to the possibility that the transforming principle might rely on DNA [80].

### 2.4. The Griffith Experiment with Macromolecules

**Figure 11:**
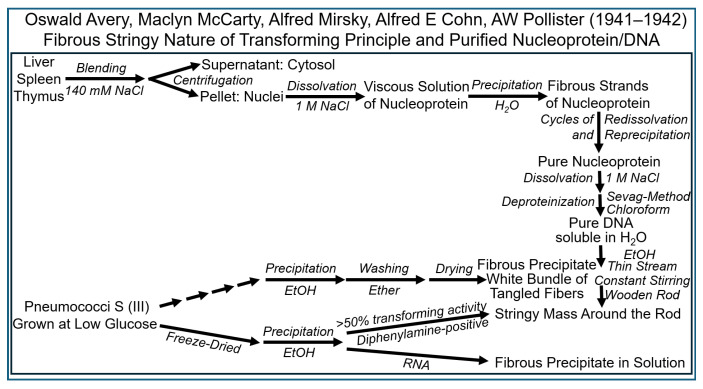
Comparative analysis of mammalian tissue and pneumococcal “chromosin” (experimental design 1.7; see Refs. [80,82]). Upper section: Organs like the liver, spleen, and thymus were blended repeatedly with physiological salt solution, which dissolved away most of the soluble materials in the case of application to pneumococci but left behind an insoluble remnant constituted largely by cell nuclei upon use for mammalian tissue lysis. The nuclear fraction was suspended in salt solution harbouring seven times the concentration of physiological salt solution. This procedure immediately resulted in the formation of a viscous solution of nucleoprotein, which was precipitated as fibrous strands upon repeated redissolvation in strong salt solution and reprecipitation by dilution. The attraction between the (very acidic) DNA and the (very basic) histones was counteracted by high concentrations of sodium and chloride ions, which necessitated the presence of levels of high salt for dissolvation of mammalian nucleoproteins. To obtain pure DNA from this mixture, Mirsky applied the same Sevag method of deproteinization by chloroform, which yielded a clear solution of DNA, irrespective of the salt concentration. In the course of precipitation of the DNA by pouring a thin stream of its solution into EtOH and constant stirring, fibrous precipitates, i.e., white bundles of DNA fibres, wound around the stirring rod, so they could be simply collected and lifted out as a single mass around the rod. Lower section: Pneumococci were grown at low glucose levels; sequentially deproteinized by EtOH precipitation; digested with Dubos SIII enzymes and RNase, extracted with chloroform; and, finally, precipitated with EtOH. After washing of the pellet with ether and its drying, the resuspended materials were poured into alcohol, leading to fibrous precipitates, which were wrapped around the stirring rod. Alternatively, some of the freeze-dried pneumococcal preparations were poured into EtOH, resulting in the formation of fibrous precipitates that were wrapped around the stirring rod and, in addition to similar fibres that floated in the solution. Most importantly, more than half of the DNA reacted with diphenylamine, and simultaneously, more than half of the transforming activity was recovered with the fraction that was wrapped around the rod. In contrast, the major portion of the RNA was left with the materials that had not been collected by the rod.

The following results raised at different laboratories in 1941–1942 hinted to the presence of thymus-like DNA, the nucleic acid-constituent of mammalian tissue nucleoproteins, in fractions of pneumococci, which display transforming activity, and “chromosin” [79,80,82] (Figure 11).

**Figure 12:**
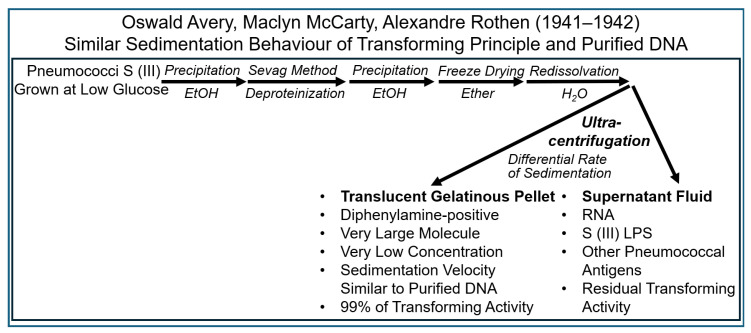
Analysis of the transforming principle using ultracentrifugation (experimental design 1.8; see Ref. [80]). Pneumococci grown at low glucose levels were sequentially fractionated in multiple precipitation, chloroform extraction and freeze-drying procedures. The fractions liberated from both protein and S (III) LPS, then redissolved in H_2_O, were subjected to preparative ultracentrifugation (30,000 rpm, 4 h). At the bottom of each of the centrifuge tubes, a gelatinous pellet was recovered after pouring off the supernatant. Upon redissolvation in 140 mM NaCl, the pellet fraction was demonstrated to display most of the transforming activity, as well as the major portion of the DNA content of the starting fraction. In contrast, most of the RNA, S (III) LPS and additional pneumococcal antigens were recovered with the supernatant.

Ultracentrifugation was introduced as a powerful tool for the analysis of biological materials based on the different rates of sedimentation, i.e., differential sedimentation velocity, of macromolecules of various sizes. The dominant feature of the instrumentation established by Alexandre Rothen is the optical system used for the monitoring of the rate of sedimentation of sample components during the centrifugation process [80]. With this apparatus, any macromolecular substance at the concentration above a certain threshold level could be detected as a moving boundary of different refraction indices while the sample of dissolved components was spinning out of solution at very high speeds. A small container was designed to enable the precise removal of individual sample components for quantitative chemical, physical and serological analyses, subsequent to the completion of the ultracentrifuge run. A large so-called concentration head allowed much larger volumes to be spun at speeds similar to those possible with an analytical chamber. Findings obtained by using preparative ultracentrifugation provided additional evidence for the operation of DNA as the transforming principle (Figure 12).

**Figure 13:**
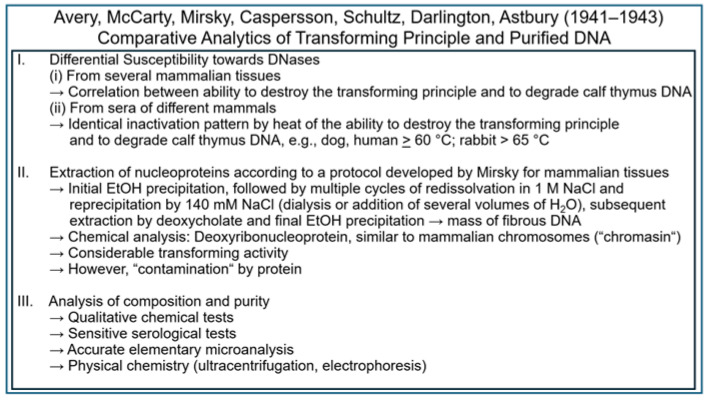
Biochemical evidence of the DNA nature of the transforming principle (experimental design 1.9; see Refs. [83,84,85,86,87,88,89,90]). I. (i) Crude enzyme preparations from various mammalian sources were incubated with crude pneumococcal fractions, as well as with DNA, which was partially purified from calf thymus. Each of the preparations with the potential to destroy the transforming activity also managed to degrade the calf thymus DNA. I. (ii) Dog, human and rabbit sera caused inactivation of the transforming activity, as well as cleavage of calf thymus DNA, which were abrogated upon heating of the sera to 60 °C and 65 °C, respectively. Thus, the inactivation pattern of the DNA-cleaving activity of the sera exactly matched that of the disruption of the transforming activity. II. S (III) pneumococci were heat-killed (65 °C, 30 min); precipitated with EtOH; washed with 140 mM NaCl; extracted for the nucleoprotein; and, finally, precipitated by EtOH. After repeated cycles of redissolving and reprecipitating, followed by extraction with deoxycholate and final precipitation with EtOH, a considerable amount of material was recovered, which harboured the expected transforming activity. III. Analysis of composition and purity by a variety of methods revealed that this material was constituted by deoxyribonucleoprotein very similar to Mirsky’s mammalian “chromosins” [82,86].

The following experimental approaches (Figure 13) were undertaken in 1941–1942 to further corroborate the putative identity of DNA and the transforming principle [83,84,85,86,87,88,89,90].

Although samples obtained by this “Mirsky” mode of preparation turned out to be still heavily contaminated by protein [80], they were important in realizing that this semi-purified DNA-containing fraction had been produced from pneumococci on the basis of a method that was completely independent of the Avery–McCarty procedure but, nevertheless, highly active in the transformation system. Thereafter, the two principal types of highly purified DNA preparation from S (III) pneumococci were subjected to rigorous analysis by a variety of biochemical approaches. In addition to assaying the final preparations for transforming activity, several different quantitative and qualitative assays were applied to assess the composition and purity of the material. Chemical tests were useful in quantitatively evaluating how successful the efforts in eliminating protein, carbohydrate, LPS and RNA had been. They were supplemented by much more sensitive serological tests in the case of the antigenic proteins and polysaccharides of the pneumococci, in general, and of their capsule, in particular. A further step was achieved by an accurate elementary microanalysis. The homogeneity of suitable preparations was investigated by elaborated methods of physical chemistry, such as ultracentrifugation and electrophoresis. In combination with the data on enzymic inactivation, the evidence seemed to be clear-cut that the transforming principle is identical to DNA. All the following experimentation performed by Avery, McCarty and MacLeod until 1943 at the Rockefeller Institute and published in 1944 [22], as well as research published during subsequent years [91,92,93,94,95,96] and by other researchers [97,98], predominantly aimed at monitoring the differential sensitivity of the transforming principle towards attack by (purified) proteases and DNases from varying sources, was designed to unequivocally confirm this educated guess.

In fact, meanwhile, the putative significance of nucleic acids was beginning to be acknowledged by the scientific community, in general (see e.g., Refs. [99,100,101,102,103,104,105,106,107,108,109,110]), and Mirsky, in particular. Finally, in 1943, the latter recognized the need for experimental designs for the unequivocal identification of the chemical basis of heredity, which had already been initiated with regard to bacteria by Griffith, Avery and coworkers more than 15 years prior [86]: “*the kind of experiment that is needed to place the chemistry of the gene on a firm basis is one in which substances extracted from the chromosomes of an organism are administered to a mutant form of the same organism which suffers from a deficiency in its germinal material*.”

Nevertheless, R.D. Hotchkiss was still having to fight heavily, also through experimentation, against some argumentation of the scientific community that trace amounts of protein contaminating the transformation reactions were ultimately causative of the positive outcome of the latter [111]. However, subsequent epochal experimentation carried out by Lederberg, Hershey and coworkers led to (almost immediate) termination of most doubts on the DNA nature of the transformation principle, demonstrating that the “protein coat” and the other “superficial apparatus” of donor bacteria or phages involved in the phenomena of chromosome transfer or infection of acceptor bacteria, respectively, are left behind in the course of transfer or infection (see below).

A synopsis of the various experimental designs of the Griffith transformation experiment, including their interpretation with regard to transferred entities and principles, the theory and type of inheritance, as well as the underlying intention, which had already been performed (1.0–1.3) and those which remain to be performed in the future (1.4–1.X), is presented in Table 1.

### 2.5. The Griffith Experiment as the Foundation of Chromosome Transfer and Phage Infection

Shortly after publication of the findings of DNA in the matter of (bacterial) inheritance [21], it became increasingly apparent that phenotypic switching between bacteria (nowadays termed horizontal or lateral gene transfer) cannot only be provoked by transformation (upon release of DNA from them), as was first demonstrated by Griffith [20]. It may also result from the transport of some heritable substance exclusively from donor to acceptor bacteria (upon intimate contact between them), a process then termed chromosome transfer between so-called Hfr (high frequency of recombination) and F^−^ (maternal) strains. Chromosome transfer, as a mode of recombination and parasexuality between bacteria, was first reported by Tatum and Lederberg [112], Lederberg and coworkers [113], and Zinder and Lederberg [114].

Initially, chromosome transfer was thought to happen upon transient fusion of the inner and inner, as well as outer and outer, membranes (Gram-negative) or PM (Gram-positive) of the donor and acceptor bacteria, leading to continuity between their cytoplasm and both the inner and outer membranes (Gram-negative) or solely the PM (Gram-positive). The cytoplasmic continuity, alone, should enable the transfer of the DNA [108]. This best explained the acquisition of typical metabolic or antibiotic markers, such as the use of lactose (Lac) and galactose (Gal) or resistance against azide (Az^r^) and phage T1 (T1^r^), from prototrophic donor Hfr wildtype by auxotrophic F^−^ mutants. For the mapping of the *E. coli* chromosome, “mating” bacteria can be separated with the aid of a Waring blender, which causes the immediate interruption of transfer, compatible with the time-dependent movement of DNA through the pili.

**Figure 14:**
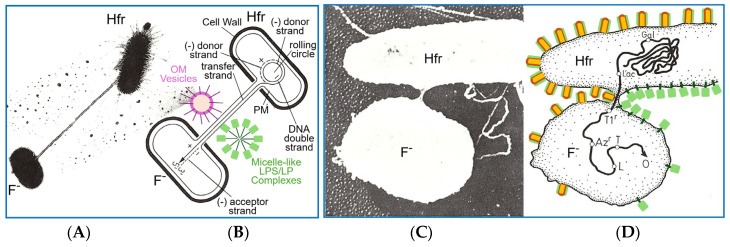
Transfer of DNA and, putatively, other matter of inheritance between donor and acceptor bacteria (**A**–**D**; adapted with modifications and permission from Ref. [115], 1973, Fritz Kaudewitz, Springer Press). (**A**) Electron microscopy of F^−^ acceptor and Hfr strains of donor cells of *Escherichia coli* K-12, as defined by low and high rates of recombination, respectively, connected by a long tube-like sexual pilus originating from the latter. The donor bacterium was decorated with appropriate fili-like RNA phages adsorbed by the cell body. For the discrimination from the *E. coli*-typical elongated thin shape, mutant acceptor bacteria exhibiting a shortened, stocky cell shape were used [116]. (**B**) Interpretation of the mechanism of the “rolling-circle” replication of DNA in the course of chromosome transfer from Hfr to F^−^ cells, which anticipates the transfer of solely the (+) strand following copying of the intact (−) strand as the template in the Hfr cell (broken line) before initiation of the transfer. In the F^−^ cell, the transferred (+) strand is supplemented with the complementary (−) strand (broken line), resulting in a DNA double helix. (**C**) Electron microscopy of a pair of Hfr and F^−^ *E. coli* cells (for their discrimination, see Ref. [117]). (**D**) Interpretation of the mechanism of transfer of DNA from *E. coli* K-12 Hfr to F^−^ cells, which anticipates the building-up of a bridge consisting of a pilus-like PM structure filled with cytoplasm connecting them.

In contrast, a function of the sexual pili, as tubes for the transfer of OM vesicles and/or micelle-like LP/LPS complexes (as indicated in Figure 14) from Hfr to F^−^ cells, remains to be demonstrated. Nevertheless, it is conceivable that in the course of “mating”, the neighbouring Hfr and F^−^ cells are prepared for local fusion of their PM or both their inner and inner and outer and outer membranes within a limited surface area. This may establish continuity of the membranes and generate a thin bridge of cytoplasm. This opens the possibility of lateral movement along the membrane plane of certain cell-surface structures, such as LPS (orange) and acylated LP (green), from Hfr to F^−^ bacteria along a declining concentration gradient of this non-DNA matter. Therefore, structures consisting of proteins and/or lipids, including OM/PM vesicles and micelle-like LP/LPS complexes, which are responsible for the replication and expression of major features and larger differences, such as morphology, rather than of metabolic or antibiotic resistance markers, could be transferred (Figure 14). So far, chromosome transfer has been based on selection for minor features or small differences encoded by DNA exclusively. This narrowing may prevent the identification of the inheritance of matter and information other than DNA.

**Figure 15:**
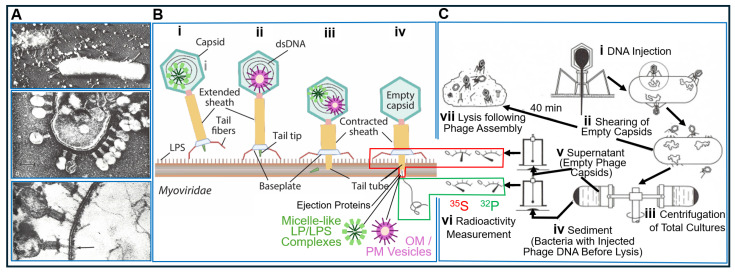
Infection of bacteria by bacteriophages for the demonstration of DNA as the matter of inheritance. (**A**) Electron microscopy (adapted with permission from Ref. [115], 1973, Fritz Kaudewitz, Springer Press). Upper section: Replication of bacteriophage *Phagus lacticola*, exhibiting a very thin and long tail structure in cells of *Mycobacterium spec.* A number of phage particles was already adsorbed by the cell. After successful injection of the phage DNA, the deeply buried phage, consisting only of the proteinaceous capsid, became easily penetrated by electrons due to the missing DNA (grey colour; see (**B**), stage iv vs. the electron-dense “white” DNA-filled capsids; see (**B**), stages i–iii). The cell in the upper-left section was filled with newly assembled phage particles due to the high phage multiplicity applied and was already prepared to undergo lysis. This led to the release of infectious phage particles. Middle section: Electron microscopy of particles of phage T4 following adsorption by *E*. *coli* cells at high multiplicity. The previously extended tail sheaths were already contracted (see (**B**), stages i/ii vs. iii/iv). Lower section: Electron microscopy of the ultra-thin section of the momentum of injection of phage DNA across the cell wall (see (**B**), stage iv), which was penetrated by the tail tube (see (**B**), stages iii and iv) upon first contact of the tail fibres (see (**B**), stage i) and tail tip (see (**B**), stage ii; recognizable if in the plane of section) with the cell surface (site of injection indicated (see arrow in (**A**)). The previously extended tail sheaths were already contracted. The injected DNA was clearly visible (see (**B**), stage iv). (**B**) Interpretation, including hypothetical explanations (see text for details). (**C**) Hershey–Chase experiment for demonstration of independent functions of the capsid polypeptides and the DNA of bacteriophage particles (adapted with modifications and permission from Ref. [115], 1973, Fritz Kaudewitz, Springer Press) (see text for details).

In addition to transformation and chromosome transfer of bacterial DNA, the infection of bacteria with phage DNA is considered an additional mechanism of parasexual horizontal or lateral gene transfer between prokaryotes. It also proved to represent an extremely important cornerstone for the acceptance by the scientific community of cases of DNA as the sole matter of inheritance. The corresponding experimental design was introduced by Alfred Hershey and Martha Chase in 1952 [118]. However, its interpretation has predominantly been based on the misunderstanding that DNA, alone, can enter the bacterial cell, an interpretation that is still shared by some older as well as current canonical textbooks as exemplified in the following (Figure 15).

Hershey and Chase [118] grew bacteria in a culture medium containing chemical building blocks labelled with both ^32^P and ^35^S. If those bacteria had been infected with phages, ^35^S was only incorporated into the proteins of the phage particles (supposedly solely capsid proteins) during their synthesis, while ^32^P was only incorporated into the phage DNA. Such double [^32^P]- and [^35^S]-labelled phages were then used to infect unlabelled bacteria. With suitable filters, it was easy to distinguish the lower-energy radiation of ^35^S from the more energy-rich radiation of ^32^P using a “Geiger–Müller” counter. Consequently, bacterial cells on whose surfaces those doubly labelled phage particles had been adsorbed displayed the radiation of both ^35^S and ^32^P (i). In the course of the experiment, suspensions of those bacteria were treated in a Waring blender, an apparatus similar to a kitchen mixer, a few minutes after adsorption of the doubly radiolabelled bacteriophages (ii). Importantly, the bacterial cells were not destroyed during this process. The DNA-free bacteriophage capsids, however, were sheared off the bacterial surface. If such a cell suspension was centrifuged in a low-speed benchtop centrifuge (iii), the bacterial cells become sedimented (iv), while the empty phage shells remained in the supernatant (v). Measurement of radioactivity yielded different labelling for the two fractions (vi): The sediment showed that of ^32^P (iv). It contained the bacterial cells into which phage DNA had been injected, since the phage capsids had been sheared off by the Waring blender. The phage capsids were left in the supernatant and were, therefore, labelled with ^35^S rather than ^32^P (vi). If such bacterial cells, which had been treated immediately after injection of phage DNA with the Waring blender, were further incubated, they supported normal phage synthesis.

Consequently, the observation that fully infectious T4 particles are released from *E. coli* cells upon penetration of solely phage DNA was subsequently interpreted as a strong argument for DNA as the only matter of inheritance (see e.g., Ref. [119]). This conviction was not substantially shaken by the awareness of Hershey, himself, of phage virion proteins that are embedded in the interior of the capsid along with the DNA. This so-called “germinal substance of bacteriophage T2”, which encompasses, in the case of the related T4 phage, non-essential internal proteins IPI*, IPII*, IPIII*, and gpalt*, is ejected from the infecting phage particle in cooperation with gp2 and gp3 and enters the bacterial cell [120]. In fact, subsequent experimentation revealed the association of most, if not all, phage particles with so-called viral ejection proteins. These are of non-structural function and highly dynamic nature and, during infection, are expelled into the host bacterium, where they become part of (the interior of) it. Ejection proteins may function by extension of the short tail, which mediates penetration of the cell envelope. This involves engagement in the ejection of the phage DNA as integral PM proteins of, typically, fewer than 200 amino acids (Gram-positive). Alternatively, ejection proteins may operate as tunnel-forming proteins crossing the periplasm or, alternatively, as 900- to 3300-amino-acid inner-membrane proteins (Gram-negative) [121,122,123,124]. Following ejection, huge conformational changes happen in ejection proteins, accompanied by threading of the DNA through the central tail tube, which is typically 2.5–3.0 nm wide. Subsequently, their refolding into a tunnel- or channel-like structure of varying stoichiometry occurs inside the phage particle. The total complex is sometimes called DNA ejectosome [121,125,126,127,128,129,130]. In consequence, the apparent failure of measurement of ^35^S-labelled ejection proteins during the Hershey–Chase experiment may be explained by the insufficient sensitivity of the applied radioactivity monitoring equipment (“Geiger–Müller” counter), in combination with the rather low amount of ^35^S-labelled proteins contained in the phage particles and low rates of their synthesis or turnover in most host bacteria.

These apparent experimental limitations and the putative theoretical considerations derived thereof were apparently neglected in support of the previous interpretation of the Hershey–Chase experiment. Typically, it served as confirmation that the materials viruses introduce into bacteria are DNA exclusively, thereby contributing to the DNA-centric view of inheritance [131,132]. Hershey and Chase, however, were much more cautious. Interestingly, they did not rule out the possibility that other (also non-sulphur-containing) matter could be transferred into the infected cells, in addition to DNA. They also wrote that “*the chemical identification of the genetic part*” of the matter transmitted by viruses remains an open question [118]. As late as in 1953, Hershey insisted that there was not enough evidence to conclude that genes are just DNA. He said biology remained divided on this issue, and he, himself, continued to suspect that protein also could play a role.

It looks like a kind of late “reparation” for him that, in addition to the ejection proteins, the phage capsid could harbour small OM/PM vesicles and/or micelle-like LP/LPS complexes. Those may have been incorporated into the capsids during prior infection of donor (host) bacteria and, then transferred to acceptor (host) bacteria in the course of the next infection cycle, with accompanying manifestation of a new phenotype in the latter (see Figure 14B). This type of transfer of certain (putatively) major features and (putatively) large differences from donor to acceptor bacteria with the aid of phage capsid as transport vehicles for bacterial OM/PM vesicles or micelle-like LP/LPS complexes resembles—from a mechanistic point of view—the (abortive or specialized) transduction or conjugation with substituted episomes (F’-duction) of minor features and small differences with the aid of phage and episomal DNA, respectively, as the transformation vehicle for bacterial genes. Furthermore, it may represent an additional mode of horizontal non-DNA-based matter/information transfer between bacteria.

However, from the perspective of the history of science, the relevance of the Hershey–Chase experiment for narrowing of the term “inheritance” to solely the transfer from donor to acceptor cells of matter or information required for the synthesis of proteins and for concomitant exclusion of the transfer of structural and cybernetic information seems to be of considerably greater importance.

### 2.6. Transformation, Chromosome Transfer, Phage Infection and the Central Dogma of Molecular Biology

The biogenesis of certain macromolecular complexes [133], such as tobacco mosaic plant virus [134,135], prokaryotic and eukaryotic signal recognition particles [136,137,138,139] or even-numbered T bacteriophages from the corresponding nucleic acid and polypeptide constituents, spontaneously occurs de novo in a stoichiometric process called self-assembly [140,141]. It does not depend on the aid of any external (proteinaceous) enzymic or (nucleotide-driven) templating components, which are not contained in the end product or final complex. Thus, T4 phages become inherited and replicated upon infection of *E. coli* cells in the course of self-assembly, i.e., the mere translation of the protein’s linear amino acid sequence and its folding into tertiary and quaternary conformations rather than self-organization [142]. According to the central dogma of molecular biology, transformation, chromosome transfer and phage infection ultimately rely solely on the transfer of information for the synthesis of proteins from one bacterial cell to the next. Transfer of structural and cybernetic information is not necessary for an adequate explanation of the inheritance of macromolecules up to the complexity of ribonucleoprotein structures and bacteriophages for the following reasons:

(i) They are not based on biological membranes and are, therefore, not faced with the problems of topology and orientation. (ii) They do not underlie metabolism and turnover, which necessitates regulatory circuits and feedback loops between system components and metabolite fluxes. (iii) They do not rely on the continuous uninterrupted growth and replication of successor structures via the incorporation of newly fabricated components into pre-existing predecessor structures in a process called self-organization [140,142]. In a certain sense, transformation, chromosome transfer and phage infection have to be considered as the inheritance of minor differences only (e.g., between antibiotics sensitivity and resistance) rather than as the inheritance of a (major) feature (e.g., acquisition of a pilus-like structure). The former is based on the necessity of transferring only the information required for the synthesis of new proteins as well as their self-assembly into new (macro)complexes rather than the structural and cybernetic information required for their self-organization, as well as their communication, interaction and cooperation with other (pre-existing) structures and metabolic fluxes of the host cell (e.g., PM transporters and ion currents).

### 2.7. Bacterial Prions as a Putative Transforming Principle

Prions are protein aggregates with the capability of self-propagation that consist of specific polypeptides adopting multiple alternative three-dimensional conformations. In mammalians, they are responsible for the pathogenesis of fatal transmissible spongiform encephalopathies and devastating neurodegenerative diseases such as Creutzfeldt–Jakob disease (for a review, see Refs. [143,144,145,146,147]. Therefore, non-pathogenic prions have been identified in a multitude of different species, including budding yeast, Drosophila and Arabidopsis (for a review, see Refs. [148,149,150]), in which they operate as proteinaceous mediators of epigenetic information. In other species and fungi, prions can also be regarded as non-toxic components of protein-based inheritance. The specific characteristics of prions are most often exerted by a module termed the prion domain (PrD), which is necessary and sufficient for the propagation and phenotypic consequences of the prion. Transformation of the soluble form to the highly structured aggregated prion form is commonly accompanied by either a loss of function or gain of function of the associated polypeptide, ultimately leading to an advantage in fitness under given environmental states. A special feature of some prions is their ability to adopt a variety of structures, termed strains, each causing their propagation with different characteristics. In both mammals and yeast, the pathogenesis of different diseases and the expression of different phenotypes, respectively, may be caused by different types of the corresponding prion protein. Although the details of the molecular mechanism of the propagation of prion proteins remain to be elucidated, preliminary investigations in mammals and yeast favour a model of nucleated polymerization. Accordingly, proteins are transformed from the soluble into the prion form in the course of elongation of pre-formed prion aggregates in the oligomeric state. In contrast, fully elongated prion aggregates are susceptible to fragmentation into smaller oligomers upon the action of Hsp104/ClpB, a chaperone exerting ATP-dependent disaggregase activity, which presumably fosters the propagation of prions in yeast. In addition, initial transformation to the prion form seems to be the result of a rare spontaneous event of oligomerization remaining under a critical size, which enables the oligomers to transform back to the soluble form [151,152].

During the past decade, a large number of cellular prion domains (cPrDs) has been found in bacteria on the basis of a bioinformatic approach [153,154]. To date, two of these cPrDs have been demonstrated to undergo self-propagation and cause the formation of prion aggregates in *Escherichia coli*, the PrD expressed in termination factor Rho of *Clostridium botulinum*, the causative agent involved in the formation of prions upon exposure to full-length Rho polypeptide [153], and the PrD encoded by the single-stranded DNA-binding protein of Campylobacter hominis [154]. Moreover, cPrDs have been predicted for additional orthologs of these proteins. Importantly, although certain bacterial lineages manage to propagate prions for a considerable number of generations (>100), a portion of bacterial cells in each lineage apparently gets rid of the prions in each round of replication as a consequence of an unknown mechanism of prion propagation in bacteria. It may or may not follow the nucleated polymerization model operating in mammals and yeast. There is experimental evidence that the loss of the prion in the labile subtype may be due to stochastic and asymmetric transfer of the prion aggregates to only one of the two daughter cells in the course of division of bacteria, leading to “partitioning errors” during the inheritance of the self-propagating prions [152].

Previously, the “PrionScan” bioinformatic approach was used to identify PrDs in 839 different proteomes of bacteria, leading to the elucidation of 2200 putative prions. This analysis argued for specific structural and functional characteristics of those polypeptides—in particular, rearrangement at the cell periphery, assembly of macromolecules, adaptability to cells and invasion of eukaryotic cells [153].

Amyloid-forming proteins considerably vary with regard to sequence, origin and function and all share the characteristics of generating ß-sheet aggregates (for a review, see Ref. [155]), with some inducing their production in response to interaction with host proteins under tight control of their assembly by the cell with the help of chaperones and spatiotemporal regulation (for a review, see Ref. [156]). It appears that cells use the production of amyloid fibrils for various purposes [157,158,159], such as the transfer of heritable information, e.g., yeast prions (for a review, see Ref. [147]). Prions form a singular subset of amyloid-like proteins, which manage to switch from one conformation to the next, often identical to amyloid aggregates, and transmit this state to distinct homologous protein species [160].

Interestingly, novel data argue for infectivity and prion-like activity of amyloid proteins in the brain, causing Alzheimer’s and Parkinson’s diseases [161]. Prion-like proteins belong to a class of aggregation-prone polypeptides with specific sequence requirements and are supported by cofactors, particularly lipid or lipid-like molecules, in triggering the protein conformation-based infectious agent [147]. Typical amyloid proteins exhibit specific areas enriched in hydrophobic amino acids inducing their self-assembly, while prion-like proteins display regions that are rich in asparagine and glutamine [162], as well as in tyrosine, glycine and serine residues [163]. These special sequence requirements correspond to sequences of low complexity exhibiting disordered structures, a critical parameter that guarantees flexibility in conformation, enables self-assembly despite the lack of necessity for unfolding of the conformation and is compatible with inter-species transformation (for a review, see Ref. [160]). Consequently, the propagation of amyloid aggregates relies on features, i.e., the extent of enrichment/deprival of specific amino acids, as well as the length of the relevant region of low complexity [163].

For differentiation between prion and non-prion amyloid proteins [164], bioinformatics approaches for the prediction of putative prion proteins have been developed. Initial predictive algorithms relied on the primary sequence exclusively, triggering the production of typical amyloid aggregates, i.e., extensive hydrophobicity and an inherent trend for ß-sheet formation). However, asparagine/glutamine-rich stretches were not monitored by this algorithm on the basis of their polar nature and the failure to support the typical aggregation associated with ß-sheet amyloids [147]. Subsequently, algorithms were developed to localize segments rich in asparagine/glutamine in the primary sequence [165] but failed to rank the proteins with regard to their relative prionogenicity. A considerable point of progress was the combination of computational approaches and experimental validation, resulting in the correct scoring of new proteins according to their in vitro prionic characteristics and permitting a big improvement in the currently available theoretical models.

Bacteria manage to adapt to considerably differing environmental conditions and to grow under extremely adverse conditions. Knowledge about the expression of so-called prionic features of bacterial proteins may support the understanding of the biology and pathogenesis of bacteria. Initially, the production of amyloids was assumed to be limited to eukaryotic cells, but in subsequent studies about the emergence of curli fibres at the surface of E. coli cells, which share physical features with human amyloids (for a review, see Refs. [166,167,168]), the number of amyloid-like bacterial proteins was found to steadily increase (for a review, see Refs. [169,170]). Finally, bacterial amyloid-like proteins can trigger the production of amyloid aggregates in response to interaction with a multitude of host proteins (for a review, see Ref. [171]). The formal similarity between DNA and protein transformation experiments led Gerald Fink to ask an intriguing question [172]: “*Would history have been different if virulence in pneumococcus had been caused by a prion and Avery et al. (1944)* (see Ref. [22]) *had discovered that the transforming principle was sensitive to a proteinase but not DNase?*”.

## 3. Perspectives and Conclusions—Thought Styles About and Reception of Transformation

### 3.1. Conceivable Alternative Explanation of the Griffith Transformation Experiment

**Figure 16:**
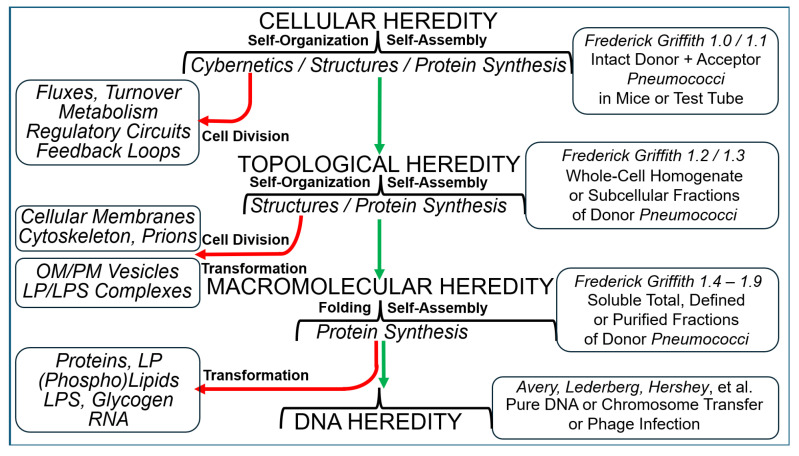
Flow chart of the scientific reductionism leading from cellular to DNA heredity via topological and macromolecular heredity (green arrows) under the accompanying sequential elimination of “cybernetic” and “structural” information in the course of cell division (red arrows), including regulatory circuits and cellular membranes, and transformation (red arrows), including OM/PM-vesicles and proteins, under concomitant narrowing of the capability of protein synthesis by the elimination of non-DNA matter (red arrow). The biogenesis of macromolecules, cellular topology and total cells critically depend on folding, self-assembly and self-organization, respectively, as indicated. The levels of the various designs of the Griffith transformation experiment (see Ref. [21]) and its successors (see Refs. [22,70,71,72,73,74,75,76,77,91,92,93,94,95,96,112,113,114,118]) on the neglect, exclusion and emphasis of the transfer of information for cybernetics, structures and protein synthesis, respectively, are indicated.

The above considerations suggest that biological inheritance, defined as the replication and transfer of matter (materials, substances) and information (shape, form) from donor to acceptor (bacterial) cells, leading to phenotypic consequences, should be extended to topological and cellular heredity rather than narrowed to macromolecular heredity (Figure 16).

**Figure 17:**
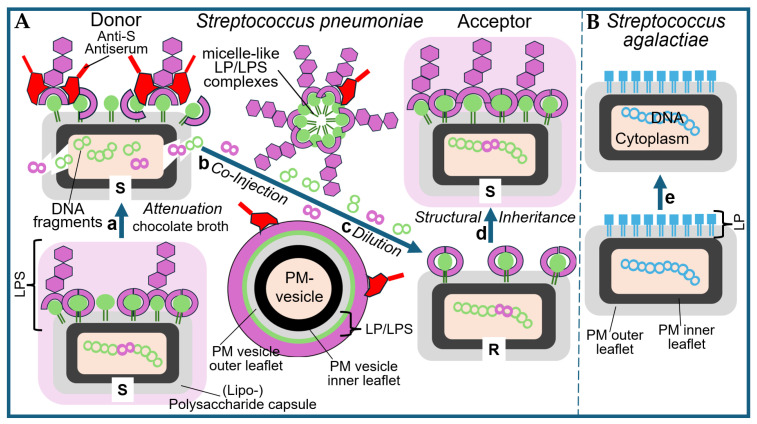
Putative role of structural inheritance for the transition of donor R to acceptor S pneumococci in the Griffith transformation experiment (design 1.0). (**A**) Donor pneumococci of serotype S display a (lipo-)polysaccharide capsule consisting of LP and LPS, which are encoded by corresponding genes (pink circles) embedded in the total bacterial genome (green circles). (**a**) Their growth was attenuated by the presence of chocolate broth and anti-S antiserum raised against serotype S. (**b**) Attenuated S pneumococci were co-injected, together with acceptor pneumococci of serotype R, into mice. The attenuation procedures led to increased susceptibility of S pneumococci to (limited) degradation and lysis, which are caused by the specific conditions of the host organism and result in the release of DNA fragments (green colour, genome encoding the R phenotype, i.e., lacking the gene responsible for LPS synthesis; pink colour, gene responsible for LPS synthesis), micelle-like LP/LPS complexes and PM vesicles with LP and LPS inserted into their outer leaflet. (**c**) It remains to be clarified whether the anti-S antiserum, which becomes diluted in the course of the injection, “actively” participates in the production of the micelle-like LP/LPS complexes and/or PM vesicles. (**d**) The released DNA fragments encoding genes for the synthesis of the S-antigen (pink circles), as well as the LP and/or LPS, are integrated into the genome of acceptor pneumococci of the R phenotype (green circles) by recombination, insertion or fusion, respectively. It critically depends on the expression of pre-existing DNA and PM with their LP and LPS constituents. The latter structures operate as a template to enable mutual interactions and the correct assembly of the newly synthesized and subsequently inserted (micelle-like LP/LPS complexes) or fused matter (PM vesicles) into the capsule of serotype S. (**B**) Pneumococci of different species, e.g., *Streptococcus agalactiae*, do not harbour LP and/or LPS (green spheres) in their PM throughout their “history” but unrelated “false” counterparts (turquoise cuboids), as well as DNA not related to the genome of *Streptococcus pneumoniae* (turquoise circles). (**e**) They are not able to acquire, replicate and transfer the new virulent S serotype.

PM vesicles and micelle-like LP/LPS complexes may operate as candidate vehicles for the transfer of matter and information for structural or templating inheritance, as is exemplified in the hypothetical model for the transformation of R to S pneumococci (Figure 17).

Considering this putative mechanism, the inheritance of structural matter, such as PM/OM vesicles or micelle-like LP/LPS complexes, should no longer be regarded as a problem of unconceived alternatives “exceeding our grasp”. This conception has previously been proposed by P. Kyle Stanford to explain the non-appearance, missing awareness or (pre-mature) disappearance of (fundamental) theories, including Darwin’s pangenesis or molecules of memory [173,174,175]. Current adequate conceptions of inheritance between donor and acceptor cells, in general, and microorganisms, in particular (Figure 16, Table 1), should include (i) PM/OM vesicles, micelle-like LP/LPS complexes and bacterial prions (for details, see above]), the inheritance of which may be demonstrated by transformation, chromosome transfer or phage infection; (ii) topological heredity encompassing the transfer of structural information, as well as PM and cytoskeletal matter (not discussed here; for a review, see Refs. [16,176,177]); and (iii) cellular heredity, including the transfer of cybernetic (or systemic) information, as well as matter constituting regulatory circuits and metabolite fluxes.

Over the past 150 years, scientific reductionism has led to the neglect or even exclusion of cybernetic (systemic), as well as structural (templating), inheritance from the analysis of cellular heredity, leaving macromolecular heredity, corresponding to protein synthesis ability, as the sole topic of scientific interest and investigation. The matter (or information) of inheritance and the question as to whether polysaccharides, proteins, RNA or DNA manage to fulfil the job remained unsolved until the midst of the last century (Table 1). During the last three decades, geneticists, as well as developmental and evolutionary biologists, have tried to compensate for those deficits with the introduction of conceptions of an “Extended Theory” [178,179,180], “Extended Evolutionary Theory” [181,182,183,184] and “Evolution in Four Dimensions” [185,186,187,188], unfortunately with limited success only. Nevertheless—or precisely because of that—the identification of human and non-human (f)actors that might have been responsible for reductionism in the past and the ongoing neglect of non-DNA matter and information of inheritance [53,54,189,190] may be of relevance for the creation of novel microorganisms using synthetic biology approaches, in general, and transformation, in particular.

### 3.2. Putative (F)Actors That Affect the Identification of the Transforming Principle

Apparently, the focus of research on inheritance between 1928, as initiated by Griffith [21], and 1952 as initiated by Hershey and Chase [118], shifted two-fold regarding (i) the underlying matter that solely resides in DNA (DNA heredity) rather than proteins and (ii) the unique relevance of the information regarding protein synthesis rather than that for structures and cybernetics (see Figure 16). The identification of the human and non-human (f)actors (causally) involved in this two-fold shift will hopefully open venues for the creation of novel microorganisms without the narrowing and consequential fixation to DNA heredity exclusively. It is and will be one of the strengths of present and future science and technology studies (STS) [191,192], in general, and of the actor-network theory (ANT) [193,194,195], as well as agential realism (AR) [196,197,198], in particular, to address the following questions:

(i) Which “agential cuts” were employed in dealing with the phenomenon of heredity (i.e., in separation into dichotomies by agency rather than assuming pre-existing separated entities as a fact; e.g., donor and acceptor bacteria, DNA and proteins, chromosomes and cytoplasm, DNA heredity and topological/cellular heredity, matter and information, and inherited and acquired traits). (ii) Which scientific, cultural, societal, historical, economic and political (f)actors (e.g., researchers, bacteria, LPS, methods, papers and readers) were engaged in the “agential cuts”. (iii) How were the “agential cuts” accomplished (e.g., experimental systems, homogenization, centrifuge and economic systems). (iv) What was the rationale, motivation or reason for the (f)actors performing “agential cuts”? The discourse on points (i–iv) may encompass—but is presumably not limited to—the following argumentation:

(**a**) Cells as a republic: The organismic cell operates, in a way, like a democratic republic with either centralistic or federal organization corresponding to DNA and chromosomes vs. cytoplasm and cell surface, as was first proposed by Jonathan Harwood [199,200]. A centralistic republic makes suggestions in a paternalistic fashion and gives clear-cut orders, in comparison to the mutual communication, interaction and cooperation of a federal republic. In the 1920s and 1930s, a heated discussion arose among German researchers of eukaryotic genetics as to the extent to which the cytoplasm plays an important role in heredity. For representatives of the so-called “nuclear monopoly” on one side, it was only the chromosomal DNA/genes residing in the cell nucleus of eukaryotes that had decisive influence on the development of each cell, while the cytoplasm provided mere raw materials for the effect of DNA/genes. For the proponents of the “plasmon theory” on the other side, the “plasmon”, i.e., structural and cybernetic inheritance according to our current understanding, clearly had to be placed above DNA-mediated inheritance. The temporal and spatial order of DNA/gene-related developmental processes would not be conceivable without the coordinating and regulating effects of a holistic overall structure and organization prevalent in the (topological and cybernetic information of the) cytoplasm (the so-called “plasmon”).

According to Harwood [199,200], neither the arguments of the “plasmon theoretics” nor those of the “nucleus monopolists” were solely based on empirical reasons, but both presupposed certain assumptions. If one tries to establish such assumptions, it is striking that the participants on both sides of the debate repeatedly made use of analogies from the social sphere. “Nucleus monopolists” presented genes as the “dominant” authority, while “plasmon theoretics” spoke of DNA/genes and the cytoplasm as “equal partners” with “equal rights”. Since analogies of a different kind and more politically neutral expressions have become available, geneticists apparently perceived the cell as a social system, albeit corresponding to fundamentally different patterns. For “plasmon theoretics”, the creation of harmony within the (genetic and societal) system is absolutely indispensable. At the cellular level, DNA/genes alone would not be able to regulate their own affairs in such a way that the organism could develop in an orderly, well-organized and structured fashion. The cell, like society, needs a “plasmon” as a harmony maker. The “nucleus monopolists” rejected those ideas. At the cellular level, there is no ordering problem, so there is no need for a “plasmon” or any other mechanism that would direct the effects of DNA/genes. Apparently, there was no harmony at the societal level in societies of those times, in general, and in the German “Weimar republic”, in particular. Nevertheless, it was self-evident that a constant conflict between the interest groups and the balance of their interests had to take place in a democratic society, i.e., between nuclear and cytoplasmic structures and their mutual interactions in a cell [199,200]. One could speculate as to whether Griffith sympathized less with the views of the “nucleus monopolists” than with those of the “plasmon theoretics” and whether, possibly for this reason, he lost interest in a deeper analysis of the transforming principle, focusing on DNA and genes exclusively.

(**b**) DNA as book of life: The metaphor of the “book of life” for the genetic code seems to be very attractive with regard to providing meaning, relevance and eternity with the aid of literacy, narratives and letters written “black on white”. Both genes (as specific sections of DNA) and books exhibit a defined beginning and end, connected by a linear one-dimensional sequence of meaningful letters, which manage to induce causes and consequences in sequential fashion [201]. Both are principally amenable to deciphering by experimental and hermeneutic methods, which, in concert, produce sense and impact. Hans-Jörg Rheinberger emphasized the unique meaning of the act of (science) writing and the (paper) product of the written [67]: “*All being as ‘being there’ is written being*”. Certainly, this statement was originally dedicated for the writing of and the written in common readable books. However, it may simply be extended to DNA and encoded information. Thus, both books and DNA (genes) make sense, produce meaning and have a function that is open for deciphering of narratives or stories and the history of individual or evolutionary development of (micro)organisms.

It is quite illuminating that within the natural sciences, questions of sense, meaning and function are acceptable within the area of biology exclusively. It is possible to ask for the function of a specific gene or the meaning of a specific DNA sequence as it is useful to analyse the sense of a certain narrative or plot represented in a book. In contrast, no physicist would ask for the meaning of the laws of gravity, and no chemist would study the sense of hydrogen bonds (not to mix up with their role or properties). Thus, the metaphor of “book of life” can only be applied to biological research, in general, and genetics studies, in particular, and, in this, regard cannot be regarded as artificial or arbitrary. Even identical termini have been introduced for the handling and processing each of these two types of books: The (epi)genetic code becomes “written”, “read” and “erased”. As holds true for DNA matter and information, books, as physical objects and mental attitudes, are transferred or copied from one reader generation to the next. And again, the linearity and one-dimensionality of DNA heredity (nicknamed “linear biology”) correspond well to simple entertaining and fictive literature but contrasts the non-linearity and complexity of the understanding of topological and cellular heredity as well as of demanding and serious literature, respectively.

(**c**) Inheritance as a socio-cultural phenomenon: Terms such as “heredity”, “inheritance”, “hereditary”, “inherited” and “hereditable” are derived from the socio-cultural and legal domain and refer primarily to the transfer of property, goods, buildings, wealth, money, etc., from parents to their offspring. Hippocrates and Aristotle never used “heredity” and related terms or their ancient Greek counterparts to explain the biological phenomenon of “like from like”. But they did use such terms to describe the socio-cultural and legal transfer of estates. Therefore, the use of those terms in the field of biology and life sciences has to be evaluated, at a first glance, as the introduction of a given metaphor for describing a biological phenomenon. Human descendants inherit goods and wealth from their parents, just as the offspring of organisms and daughter cells inherit certain traits, differences and features from their parent organisms and mother cells. respectively. In the socio-cultural environment, “heredity” means the process of transfer and acquisition of property via the process of donation–acceptance among people who have a (close) biological or social relationship at the individual level. In the biological context, “heredity” is the process of the transfer and acquisition of traits by organisms in a biological lineage. Thus, the concept of biological inheritance is a typical example for the adaptation of a metaphor [66,202]. But the use of this metaphor has been, for the past 150 years, at least, so self-evident and “automated”, i.e., without any reflection, that hardly any notice is taken of its origin [203].

The important point relevant for interpretation of the Griffith transformation experiment (1.0) is that the daily socio-cultural and legal use of the term “inheritance” is typically restricted to the transfer of physical entities and materials (money, houses, etc.). It does not consider any (infra)structure required for the realization, implementation and enforcement of the corresponding transfer processes, such as district and probate courts, notaries, appropriate regulations, rules and laws of heredity, land registries, etc. The same holds true for any (mental) views, (basic) convictions and (common) attitudes prevalent and dominant in society, which provide the philosophical, sociological, political, legal, economic, and historical background, as well as the prerequisite for the acceptance of the phenomenon of inheritance of wealth by the members of a society. Acceptance may be independent of whether its realization is of advantage for the individual or not. Thus, the emphasis on the transfer of matter (DNA and money, buildings, etc., respectively) and the neglect of the transfer of information (topology of structures and cybernetic control of metabolism and courts, notaries, regulations, laws, etc., respectively), including the willingness of the (scientific and social) community to accept them, is shared by the phenomena of cellular and socio-cultural heredity [203,204].

Furthermore, the use of the terms “heredity” and “inheritance” for biological and socio-cultural processes represents a case for the transition of a phenomenon from the public to the scientific domain. Rather early in molecular research on biological inheritance, i.e., at the end of the 19th century, E.B. Wilson stated the following in the first edition of his remarkable book on “The Cell in Development and Heredity” [205]: “*Inheritance is the recurrence, in successive generations, of like forms of metabolism; and this is effected through the transmission from generation to generation of a specific substance or idioplasm which we have seen reason to identify with chromatin”*. In a different context, he concluded, in 1895 [206], that “*inheritance may perhaps be effected by the physical transmission of a particular chemical compound from parent to offspring*”. Thus, for Wilson, the critical point of inheritance seemed to be the donation, by donor or mother cells, of a physical substance, such as chromatin or nuclein, which, after transfer, undergoes acquisition by acceptor or daughter cells. It is even conceivable that Wilson tried to integrate a piece of the conception of cybernetic information into his interpretation of inheritance, concerning his reference to “like forms of metabolism”.

Wilson was not the only one among the early (molecular) geneticists who failed to acknowledge the necessity of providing an explanation for the initial process prior to transfer and donation–acceptance, that is, Wilson did not take into account the mechanism causing the copying or replication of a specific feature as well as its physical basis, which operates for an indefinite number of cycles of multiplication over successive generations. Wilson and his colleagues only took into consideration the “given” of the “transmission” of a feature or material and presupposed that it would be accurately reproduced with the aid of some mechanism. Apparently, its elucidation did not attract their interest. It is likely that those geneticists were not completely aware of the requirement that the chemical substance recognized by them accumulate (as a consequence of its synthesis) before it can be donated and accepted. The tackling of this question was not defined as an urgent problem.

Apparently, the same held true for Griffith. Inheritance was perceived as the transmission of a very small replica of the parents to the children, e.g., as a homunculus in the head of a sperm or yolk of an ovum. This self-reproduction was considered to be simply inherent in the sperm or ovum and interpreted in a vitalistic fashion as a natural and typical characteristics of life, as a self-evident phenomenon nor requiring need of further explanation. The apparent self-evidence of an automatism for the multiplication of inherited matter in biology may be induced or subsequently fostered, at least by analogy with the common conception of socio-cultural heredity. In fact, there is no need for the multiplication of wealth prior to its donation by parents and acceptance by children (although, certainly, the possibility of its multiplication, per se, is welcomed). Thus, the emphasis of the early geneticists, including Griffith himself, on the transfer step encompassed the identification of the nature of the transferred matter but concomitantly neglected interest in the elucidation of the mechanism of its replication.

Finally, to complete this point, the idea of a “self-replicating” molecule emerged rather late after the introduction of experimental transformation and was first addressed by John B.S. Haldane in 1937 [207]: “*Two possibilities are now open. The gene is a catalyst making a particular antigen or the antigen is simply the gene or art of it let loose from its connection with the chromosome. For one essential property of the gene is that it reproduces its like at each nuclear division… Since a mutated gene is reproduced in its altered form it follows that one gene is reproduced from another. This is an exceptional situation. A molecule of haemoglobin in a cell is not derived from another similar molecule. If it were, slight changes in the molecule would be perpetuated, and extra-nuclear inheritance would be as common as it is actually rare. But one gene is derived from a like gene… It must on the contrary, be a process of copying. The gene, considered as a molecule, must be spread out in a layer one ‘Baustein’ deep. Otherwise it could not be copied. The most likely method of copying is by a process analogous to crystallization, a second similar layer of Baustein being laid down on the first… But we could conceive of a process analogous to the copying of a gramophone record by the intermediation of a ‘negative’ perhaps related to the original as an antibody to an antigen. The process normally stops when one copy has been made, or at least the further copies are not attached to the chromosome.*”

Thus, the perception by early geneticists of inheritance as the transfer of wealth between individuals (of Western society) strongly supports a flux of knowledge (e.g., legal prerequisites, types of inheritable matter and modes of copying) from the public domain (general or layman knowledge) to science (expert knowledge). This type of flow of knowledge from lay people to experts was first acknowledged by Ludwik Fleck [208], the great philosopher of science, founder of the sociology of science, physician, bacteriologist and immunologist. He claimed that changes in the thought style in the “reverse” direction (since, typically, lay people are thought to be informed by experts only) had happened multiple times in the past and still in almost any field of Western natural science. Fleck postulated an internal structure of the thinking collective based on the particularly important distinction between an esoteric circle of specialists and an exoteric circle of interested laymen. There may be multiple gradations between these two extremes. In Fleck’s view, the internal structure of the “Denkkollektiv” corresponds to various forms of written publication, i.e., journal science (papers), handbook science (reviews), textbook science (settled wisdom) and popular (non-fiction) science (general knowledge). Whereas it is commonly accepted that (only) the esoteric circle exerts an effect on the exoteric periphery, the conception that the exchange of ideas between the circles goes in both directions is less common. Nevertheless, it is often hard to delineate the relative contributions and effects of expert and popular science on the resulting world view in terms of cause and consequence. Moreover, it is sometimes impossible to discriminate between expert and popular science due to their mutual interdependence and interactions with regard to their origin, tradition and agency in the past and present. According to Fleck [209], popular science “*forms the specific public opinion and the world view and in this form has an effect on the expert*”.

(**d**) Egoisms and selfishness vs. cooperation and empathy: The “reversed flux” from the public to the scientific domain with regard to the emphasis on competition, struggle and exclusion might also be reflected in the thought style of the DNA/gene/chromatin/nucleosin/nucleus acting as the egoistic and “strongest as the most powerful alone” (f)actor in biological inheritance vs. cooperation, coordination and inclusion operating as inheritable socio-cultural (f)actors in policy, economics and society, i.e., as a desired driving force, at best. The thought style of “social Darwinism” emerged during the last third of the 19th century and continued in the first third of the 20th century under the accompanying emergence of the libertarian or capitalistic economical systems in the Western world with the aim of the accumulation of money and wealth, per se, as private property, as the replication of DNA represents “egoistic”, “parasitic” and “private” aims, per se. These “strategies” of acquisition and multiplication of private wealth and DNA, respectively, necessitated the neglect of “peripheral” social and economic obligations of the society and cytoplasmic (non-DNA) elements of the cell, respectively, and, concomitantly, of the need for or meaning of cooperation between the societal/economical and cellular constituents, respectively.

Therefore, it is all the more surprising that, despite the prevalence of a deep economic crisis in the United Kingdom during the 20s and 30s of the last century, Griffith attributed to bacteria “altruistic” rather than “egoistic” behaviour. The principle of survival of the fittest in biological evolution at the level of populations or species could have been applied to prokaryotes as well. A bacterial cell (of a given population or species) is prepared to transfer a component of itself to a neighbouring bacterial cell (of the same population or species) as a (beneficial) “pabulum” or “signal” at the expense of its own structure, metabolism or integrity. The donation of such a “pabulum” or “signal” could be considered a coordinated, cooperative and inclusive action. As such, it totally differs from the process of transfer of the mere information of protein synthesis to acceptor bacteria, which is capable of indefinite reproduction without major consequences for donor bacteria. This could be interpreted as a reflection of the welfare state, which is based on a social market or socialistic economic system. Interestingly, during the period of Griffith’s transformation experiment, the political foundation of the Western welfare state was heavily criticized and attacked by parties of the right-wing spectrum with their nationalistic and egoistic attitudes.

(**e**) Biotechnological applications: The options of the prediction, control and manipulation of a variety of different biotechnological and medical applications, which encompass but are not limited to synthetic and systems biology, personalized and precision medicine, forensic DNA phenotyping, gene testing for everybody, eugenics and transhumanism, can be fulfilled best with DNA as the sole matter of inheritance. This assumption is based on the linear one-dimensional arrangement of letters encoding the information required for the synthesis of proteins in the “book of life” (see above), which is easily amenable to analysis (by sequencing) and change (by recombinant gene technology). In contrast, topological and cellular heredity, as manifested in the structural and cybernetic information for the three-dimensional configuration and interaction of macromolecular complexes, membranes, regulatory circuits, feedback loops and metabolic fluxes, is considerably more resistant to prediction, control and manipulation. The expenditure for it may be reduced in the future with the aid of systems biology, however, certainly will never be as low as that for DNA.

(**f**) Causal vs. explanatory sufficiency: The emphasis on DNA heredity vs. the neglect of structural and cybernetic heredity may be understood as the distinction between explanatory and causal sufficiency. Certainly, the transfer of information for the synthesis of proteins is not causally sufficient to explain growth and mitosis of cells, since, admittedly, DNA, per se, represents a “dead” molecule incapable of driving cellular life. Nevertheless, the replication and transfer of DNA are commonly accepted to be explanatorily sufficient for the phenomena of topological and cellular heredity. However, for those two phenomena to causally happen, the inclusion of structural and cybernetic information is certainly necessary, i.e., a conditio sine qua non. However, these obvious causal relationships and mechanistic links may be regarded as trivial and self-evident rather than as useful and enlightening [210,211,212].

(**g**) Form vs. substance: All organisms are exposed to metabolism or turnover. In fact, the continuous and life-long exchange of matter or substance, i.e., atoms, molecules, complexes, structures and cells, under concomitant maintenance of their shape or form has been claimed as a critical characteristic of life. The continuity and preservation of shape or form are guaranteed by the replication and expression of the information relevant for their synthesis and biogenesis rather than solely the transfer of matter. Consequently, DNA has been commonly accepted as the information relevant for both synthesis and shaping of proteins and protein complexes in the course of folding and self-assembly (see Figure 16) [133,134,135,136,137,138,139]. In contrast, maintenance of the shape or form of membranous (sub)cellular structures and intact cells undoubtedly must appreciate the inheritance of structural and cybernetic information to enable their self-organization [140,141,142]. This problem is typically addressed only in textbook and handbook science, if at all, covering the biogenesis of membranes and organelles [213,214].

(**h**) Biopolitics: From a biopolitical point of view, only the maintenance or change of identity vs. non-identity and self vs. non-self of organisms belonging to the same species (as defined by the possibility of–either horizontal or vertical–gene transfer), i.e., of small differences or minor features, is of considerable interest. It relies on the possibility of their principal predictability (e.g., by pedigree analysis), controllability (by natural selection or breeding) and manipulation (by random or site-directed mutagenesis). This holds true for organisms ranging from bacteria (e.g., R and S forms of *Streptococci pneumoniae*) to humans (e.g., belonging to different generations, families, clans, populations, races or nations). These subtle differences and minor features are often comprehensible, conceivable and manageable (e.g., as discrete Mendelian units), in contrast to large differences and major features between different species (e.g., human vs. turtle) or different developmental stages within a given species (which are based on completely distinct building plans, such as butterfly chrysalis, larva, adult).

In fact, at the end of the 19th century, the disciplines of genetics and developmental biology were separated to follow the paths of analysing either the inheritance of small differences or the emergence of major features, respectively [215]. Undoubtedly, this dichotomy ended up in a great success, with the identification of DNA as the matter of inheritance, but simultaneously led to the neglect of the transfer of structural and cybernetic information. The biopolitical interest in the inheritance of subtle differences and minor features rather than of large differences and major features may be characteristic of Western societies. They pronouncedly trend towards individualism, i.e., an ethical thinking or value system and political philosophy that put individuals rather than collectives or societies at the centre of consideration. A primary aim of individualism in Western societies, human enhancement, is the physical and/or psychological optimization and improvement of the individual or members of a small collective compared to other individuals or collectives. It is thought that this may be achieved with the aid of biotechnology, recombinant gene technology, individualized and precision medicine, forensic DNA phenotyping, gene testing, sequencing for everybody, etc. [216,217,218]. These efforts predominantly rely on the inheritance of DNA as the matter for prediction, control and manipulation rather than on that of structural and cybernetic information, which, so far, seem to be out of reach for biopolitics in the short term due to economic, political and ethical restrictions.

(**i**) Contingency of life: The emphasis on DNA as the matter of inheritance, with concomitant neglect of the inheritance of structural and cybernetic information, could be explained by the conception of mastering contingency and making sense of life [219,220].

Its religious version interprets the “bodily” parts of a cell, i.e., cell surface, compartments and cytoplasm, in analogy to the human body, or “Leib”, whereas the brain or soul corresponds to the DNA (or nucleus). The latter, in a certain sense, is commonly regarded as “immortal”, since it is continuously transferred from one generation to the next, thereby stably determining the features of the body through generations. In short, DNA seems to act as a representative of God.

The atheistic version of the above conception interprets DNA as immortal, too, but as a “parasite” of each cell. According to Richard Dawkins [221], the cell guarantees the replication of numerous copies of a DNA molecule for the sake of “egoistic” survival and the immortality of DNA itself. He does not consider DNA as mere information source for the “ordering” of proteins by newly emerging cells, as proposed by Alfonso Martinez Arias [188]. In addition, the fact of the almost perfect replication of DNA (considering the various sophisticated mechanisms of its repair), in combination with its apparent resistance to adverse effects exerted by the environment (e.g., UV light), could be regarded as an additional explanation for the apparent admiration to “adoration” of DNA. In contrast, the cell (or the body) is typically exposed to a multitude of diverse environmental factors causing transient (non-hereditary) alteration in its physiology and morphology. This susceptibility to exogenous influences may have led to a downranking of topological and cellular vs. DNA heredity. This discrimination between DNA and the other cellular/bodily constituents may be analogous to the separation of germline cells, corresponding to DNA, and somatic cells, corresponding to cytosolic and membranous components, as was postulated initially by August Weismann [222]. Consequently, DNA or, at his time, “idioplasm” was suggested to be continuously transferred through cell division as a sort of “Weismann substance” at the (intra)cellular level.

Taken together and irrespective of a religious or atheistic background, DNA (and the nucleus) could be considered as the inert, inalienable centre of the “spiritual”. It is separated from the rest of the “cellular world” as the “secular”, thereby resisting “mudslinging” and other unfavourable effectors. In line with Fleck (see above), scientists, in general, and geneticists, in particular, act as human beings, too, susceptible to the desire to master contingency. Inheritance, as a topic, may be of value with respect to this aim, based on its association with the immortality of germline cells and DNA and its inscription in the “book of life”.

Philosopher Vilem Flusser strongly emphasized the importance of various media, including language, metaphors, analogies and pictures, for the mastery of contingency in human life [223]: “*Human communication is an artifice whose purpose is to make us forget the brutal meaninglessness of a life condemned to death*”. The preference for the “book of life” vs. more complex, three-dimensional and networking systems in order to understand the phenomenon of inheritance may be owed to the exceptional importance of language, symbols and codes, including the genetic code. Symbols and letters are intended to represent the world, structure it and provide orientation. However, according to Flusser, symbols are dialectical in nature [223]. A symbol not only has the potential to represent or to act as the (life) world, but it also has the tendency to cover the latter. A code, as system of symbols, could obscure the (life) world more than it signifies it.

(**j**) Economical and political dichotomy in the first half of the 20th century: During the first half of the 20th century, after the founding of the Soviet Union by Lenin in 1917 as a consequence of the October Revolution, there was a first peak in the dichotomy between the well-established Western bourgeois industrial capitalist and revolutionary Eastern socialist economic systems. Capitalists accept the emergence of individual differences and social inequality, such as with respect to talent or possessions, as a consequence of the “lottery of life”. Principally, the beginnings of life cannot be planned with regard to being born at the right time, in the right place, under the right conditions in order to face and master the subsequent challenges of life under competition for limited resources. In contrast, socialists rely on fundamental predictability, controllability and manipulability in order to compensate for individual differences and social inequality, as well as to control and to steer the distribution of limited resources among cooperating human beings in a planned economy. Capitalism is based on (social and economic) evolution through variation of (social and economic) relations and selection by the free forces of the market. Socialism depends on evolution through the direct effect of the prevailing conditions, which are planned and managed by an elite, with monitoring and verification of the success of the measures. On a biological level, this corresponds to evolution through mutation and selection (Darwinism) or through the inheritance of acquired traits (Lamarckism). In the 1920s, Lamarckism was a debatable alternative to the synthetic theory composed of Charles Darwin’s theory of evolution and Gregor Mendel’s theory of heredity in research circles which were concerned with biological heredity (for a review, see [224,225]). Austrian zoologist Paul Kammerer [226,227] and Soviet Russian plant geneticist Trofim Lysenko [228,229] are prominent but also controversial representatives of Lamarckism. They were convinced that the exposure of animals and plants, such as their preferred study objects, fire salamanders and winter wheat, to extreme environmental conditions, such as humidity and cold, respectively, causes the development of certain phenotypes, such as calluses and resistance to cold, respectively, which are stably inherited over generations. The different scientific views on biological heredity found their political counterparts in the ideological confrontation of the capitalist and socialist systems in the first half of the 20th century and their extremes of “social Darwinism” and “Lysenkoism”, respectively, which experienced drastic sharpening as a consequence of the so-called “Cold War” during the second half of the 20th century.

Moreover, it is tempting to speculate as to whether human and non-human (f)actors of the frequent and severe crises of the economic and political systems that shook both the Western and Eastern World between 1923 and 1962, among them two World economic crises, the rise and fall of dictatorships in Nazi Germany and the Soviet Union, the consequences of World War II (WWII) for the transient and permanent victors and vanquished, the switch to a war economy in the United Kingdom during WWII, Churchill’s efforts to introduce a biological weapon based on anthrax toxin and the propagation of *Bacillus anthracis* for his fight against Nazi Germany, a number of so-called “proxy wars” between USA and Soviet Union, including the Korean and Vietnam wars and the “Cuba crisis” after WWII, caused the emergence and preference of nucleus monopoly, DNA-centric and macromolecular heredity vs. plasmon theoretic, poly-matter network and topological and cellular heredity conceptions in genetics research and by geneticists, respectively, of this period (see above (a)). Thereby the agency of the political and economic systems became manifested in the genetics domain as the apparent (and at least transient) superiority and strength of autocratic and totalitarian vs. democratic and libertarian systems, resembling Fleck’s reversed flow of (scientific) opinion from the exoteric periphery to esoteric cycles (see above (c)). The identification of those putative (f)actors with agency on Griffith and his successors remains a desideratum of future analysis with the aid of STS, ANT and AR.

Furthermore, the synthetic theory represents an extreme reductionism through which the diversity of the organismic world is ultimately traced back to a class of (linear) molecules (polymers) in a “one-dimensional” way. Complex structures and cybernetic feedback loops between them are not required for this. At the same time, this also largely eliminates the possibility of the agency of (f)actors of the (natural and cultural) environment, which affect cellular and topological heredity and, concomitantly, mediate the inheritance of acquired traits. Rather, macromolecules responsible for heredity must remain hidden from direct access by the environment in the depth of the cell and protected by its chemical nature in order to be able to fulfil the function of a “Weismann substance” at the (intra)cellular level.

Thus, the impossibility or possibility of hereditary environmental changes presupposes the strict separation or inseparability of genotype and phenotype, respectively. On one side, the former is guaranteed by the synthetic theory according to the chemical nature (DNA), localization (nucleus) and one-dimensionality (i.e., template for the synthesis of proteins) of the information transferred from donor or mother cells to acceptor or daughter cells. On the other side, the theory of the inheritance of acquired traits dispenses with such a separation and is, instead, based on the intercellular transfer of spatiality (i.e., material and information for structures and cybernetics). It is precisely this lack of a conceptual and substantive distinction between genotype and phenotype that Griffith was accused of by later geneticists (see, for example, Lederberg [20]). But this does not necessarily have to be understood as a weakness in the interpretation of his transformation experiment. Environmental factors such as “pabulum” or “signal” would be “inherited” as structural and cybernetic matter and information, eliminating the dichotomy between genotype and phenotype. This dichotomy of the opposite or exclusion of one or the other extreme is now viewed critically as explanation not only for cultural but also natural phenomena. Nevertheless, starting in the 1990s, the distinction between genotype and phenotype once again began to gain central importance, in general, and in the area of epigenetics, in particular (for a review, see Refs. [230,231,232]).

In conclusion, a multitude of socio-cultural (f)actors, the causal relationships between them (if any remain a matter of intense debate) have been elucidated with the aid of studies of the philosophy, sociology and history of science, in general [208,209], and ANT, AR and STS, in particular [191,192,193,194,195,196,197,198]. They have contributed and still contribute to prevention of the downranking, neglect and exclusion of cellular and topological heredity and their separation from DNA heredity. Moreover, STS analysis of their role in the stable inheritance of acquired traits, as well as under a neo-Lamarckian view on the nature–nurture dichotomy [62,225], may be of even greater importance. Cybernetic and structural forms of information, as the basis for cellular and topological heredity, respectively, are exquisitely sensitive to reconfiguration and reshaping by environmental (f)actors. The application of some of them, such as nutrition or mechanical forces, in the creation of microorganisms could result in the induction of major features and large differences vs. solely subtle characters and small deviations by random or site-directed mutagenesis of DNA or de novo synthesis of complete genomes [2,4,9].

### 3.3. Putative (F)Actors of Griffith’s Disinterest in Identification of the Transforming Principle

Griffith’s efforts with respect to bacterial transformation resulted in only a single-author publication (albeit encompassing 46 pages) on designs 1.0 and 1.1 in 1928 [21]. Undoubtedly, this work was of exceptional high quality, given the precise description of the methods used, the accurate presentation of the obtained experimental results and their very cautious and reserved interpretation. After the appearance of the paper, he terminated experimentation on transformation, thereby circumventing the necessity for a two-fold agential cut, i.e., (i) DNA from non-DNA matter and (ii) DNA from structural/cybernetic information, with the aid of designs 1.2–1.9, as introduced by his successors from 1928–1944 (see Table 1). His rationale and motivation can only be speculated upon:

(**a**) Griffith succeeded, for the first time, in the experimental demonstration of the occurrence of horizontal gene transfer between bacteria. At the time, this had been regarded as a theoretical possibility only in the microbiological scientific community, and he, himself, was very excited about the findings and, at first, could not believe them, as he stated in his publication [21]. Griffith was physician and microbiologist with special education in immunology and serology who laterally entered the field of (bacterial) genetics. He spent more than 30 years of his academic career researching the epidemiology of infectious diseases, first focusing on the classification of tubercle bacilli and later on the typing of haemolytic streptococci, salmonellae and staphylococci. Regarding the propagation of lobar pneumonia, a major health issue in the Western world in the first half of the 20th century, the discovery of the apparent “instability” of pneumococci and their conversion from R to S forms just by sharing the same environment was of extraordinary relevance, at least from the point of view of basic science. As Graham Wilson communicated (for citation, see Ref. [62]), Griffith was totally convinced of the exquisite stability of bacterial species, at least insofar as the tubercle bacteria had been considered. Nevertheless, the transfer of this newly gained knowledge to medical application, e.g., for the development of novel serotype-specific antisera for immune therapy against lobar pneumonia was less evident. This could have been the critical point for the apparent shift of his research focus from the elucidation of the material/mechanistic basis of the transformation phenomenon to aspects of applied microbiology and serology, such as the introduction of a novel classification system for the various pneumococcal serotypes. He was interested in medical research and therapeutic progress exclusively and never re-entered the area of the transformation phenomenon, nor did he refer to it in scientific talks with researchers at meetings or congresses.

(**b**) Griffith was not interested much in the transformation phenomenon he had discovered, since he did not recognize its relevance or meaning, despite the fact that he was well aware of the biological role of horizontal gene transfer in the propagation of virulent strains (see above). His apparent non-interest in the identification of the matter underlying the transforming principle may be highlighted best by a statement of the great German physicist and founder of molecular biology, Max Delbrück, about the need to determine the nature of it: “*If the genes were not encoded in proteins, then some other stupid molecule carries the genetic specificity*–*so what*?” In extension of this indifference, S.D. Elliott remembered (for citation, see Ref. [62]) that Griffith had told him the identification of the transforming principle was now “*up to the chemists*”. At the time, there was only limited interest in this topic, in general. Principal knowledge about the transfer of some materials between bacteria accompanied by some phenotypic consequences seemed to be sufficient. M.R. Pollock, who gave the Third Griffith Memorial Lecture in 1970, pointed out [62] “*that Griffith seemed to have had little idea of how this transformation came about, nor even of its great and ultimate significance. His discovery was a classic instance of pure serendipity*”, an opinion shared by other scientists [63,233,234].

(**c**) Griffith stopped experimentation due to technological or methodological problems [235]. After the initial discovery of the transforming principle and its publication, he made efforts to prepare cell-free extracts (whole-cell homogenates) from donor pneumococci. Thus, ultimately, there was some interest left, at least, to get a little bit more information about the nature of the transforming principle. Unfortunately, he failed, presumably due to the use of (too many) cycles of freezing and thawing of the donor cells as the only method available for him for the preparation of cell-free extracts. Nevertheless, Griffith was most likely aware of the principal possibility of identifying the material basis of the transforming principle, although he personally lacked motivation to spend great efforts in doing so. In fact, his successors were luckier in achieving this (see above).

(**d**) Griffith stopped experimentation due to conceptual considerations. The failure to generate cell-free extracts from donor pneumococci under concomitant maintenance of their transforming activity was interpreted by Griffith as a putative need for passage of the transforming principle and/or the acceptor cells through intact mice. Moreover, the requirement for intimate contact or extensive exchange between intact donor and acceptor cells in a process can be speculated upon, which possibly only happens as a consequence of their blending, mixing or fusion. At the time, it was unclear whether the transition of pneumococci from R to S depends on the synthesis of a specific LPS (considered, per se, as a candidate for the transforming principle). Griffith could have assumed that the putative transforming principle, such as that of LPS, becomes lost in the course of the preparation of cell-free extracts, making identification of its chemical nature difficult or even impossible. Moreover, homogenization may affect the overall structure and topology of donor cells, such as their cytoskeleton and cell surface, and concomitantly disrupt the integrity of regulatory (feedback loop) circuits and metabolite fluxes.

Possibly, Griffith could have felt (on a “rational basis”) that inheritance is more than just the transfer of the transforming principle, irrespective of whether protein or DNA. In this respect, he should be regarded as an early representative of the theory of “extended heredity” (for a review, see Refs. [178,179,180,181,182,183,184,185,186,187,188]). It is conceivable that Griffith would have agreed to the statement that acknowledged biochemist Gowland Hopkins made in a lecture at Harvard in 1936 that [236] “*so long as [the modern biochemist’s] analysis involves the isolation of events, and not merely of substances, he is not in danger of so departing from reality that his studies have no longer biological meaning*”. Indeed, two years later, Hopkins even rejected consideration of chemical structure as the basis for the explanation of morphology [237]. The putative open-minded, non-exclusive, holistic and integrative view of Frederick Griffith may have paved the path for an adequate understanding of the phenomenon of inheritance, in general, and to the creation of novel microorganisms, in particular (for further discussion, see Ref. [16].

## Figures and Tables

**Table 1:** Design of and consequences of the various Griffith transformation experiments. The various designs, which have either already been performed (1.0–1.3) or still remain to be performed (1.4–1.X, tbd) are compiled with regard to the experimental system and fractionation procedure used, the analysed phenotype, the detection method and representative authors involved. The nature of the entities and principles transferred from donor to acceptor bacteria and the theoretical bases are given. The inheritance of phenotypes that are explained best by the various experimental designs are indicated on an intentional basis. Boxes in light blue and red colours indicate the entities transferred following experimental designs 1.0–1.3 and 1.4–1.X, respectively.

Experiment	Model	Fractionation	Analysis	Detection	Author	Transferred Entities	Transferred Principle	Theory of Inheritance	Inherited Phenotype	Intention
*Griffith 1.4–1.X*	Homogenate	- Phospholipase - Centrifugation (high speed)	- Resistance - Auxotrophy	- Selection	tbd	- Prions - Int Disord Prot - OM-Vesicles - Micelle-like LPS Compl.	- Substance/Matter	- Including - Holistic	- Minor Features - Small Differences	- Mode of Inheritance
*Griffith 1.3*		- Chloroform - Centrifugation - Nuclease - Protease - Water-Sol. Fr.	- Virulence	-Microscopy - Serotypes	Avery MacLeod McCarty Alloway	- DNA	- Shape/Information	- Centric		- Novel Cells
*Griffith 1.2*		- Heat - Soluble Fract.		- Serotypes	Dawson		+	- Excluding		- Matter of Inheritance
*Griffith 1.1*	Mouse	- Heat			Griffith Neufeld Levinthal		- Substance/Matter	- “Hard“		
*Griffith 1.0*		- none		- Lethality Mice	Griffith					- Bacterial Variability - Infections

## Abbreviations

The following abbreviations are used in this manuscript:

ANT	Actor-network theory
AR	Agential realism
LP	Lipoprotein(s)
LPS	Lipopolysaccharide(s)
OM	Outer membrane(s)
PM	Plasma membrane(s)
R/S	Rough/Smooth pneumococcal serotype
STS	Science and technology studies
